# Supplementary material for: Genetic factors associated with prostate cancer conversion from active surveillance to treatment
Source: HGG Adv. 2021 Nov 19;3(1):100070. doi: 10.1016/j.xhgg.2021.100070 (PMC8725988; doi:10.1016/j.xhgg.2021.100070)
Supplement: Document S1. Figure S1 and Tables S1–S5 [file mmc1.pdf]

HGGA, Volume *3*

Supplemental information

Genetic factors associated with prostate cancer conversion from active surveillance to treatment

Yu Jiang, Travis J. Meyers, Adaeze A. Emeka, Lauren Folgosa Cooley, Phillip R. Cooper, Nicola Lancki, Irene Helenowski, Linda Kachuri, Daniel W. Lin, Janet L. Stanford, Lisa F. Newcomb, Suzanne Kolb, Antonio Finelli, Neil E. Fleshner, Maria Komisarenko, James A. Eastham, Behfar Ehdaie, Nicole Benfante, Christopher J. Logothetis, Justin R. Gregg, Cherie A. Perez, Sergio Garza, Jeri Kim, Leonard S. Marks, Merdie Delfin, Danielle Barsa, Danny Vesprini, Laurence H. Klotz, Andrew Loblaw, Alexandre Mamedov, S. Larry Goldenberg, Celestia S. Higano, Maria Spillane, Eugenia Wu, H. Ballentine Carter, Christian P. Pavlovich, Mufaddal Mamawala, Tricia Landis, Peter R. Carroll, June M. Chan, Matthew R. Cooperberg, Janet E. Cowan, Todd M. Morgan, Javed Siddiqui, Rabia Martin, Eric A. Klein, Karen Brittain, Paige Gotwald, Daniel A. Barocas, Jeremiah R. Dallmer, Jennifer B. Gordetsky, Pam Steele, Shilajit D. Kundu, Jazmine Stockdale, Monique J. Roobol, Lionne D.F. Venderbos, Martin G. Sanda, Rebecca Arnold, Dattatraya Patil, Christopher P. Evans, Marc A. Dall'Era, Anjali Vij, Anthony J. Costello, Ken Chow, Niall M. Corcoran, Soroush Rais-Bahrami, Courtney Phares, Douglas S. Scherr, Thomas Flynn, R. Jeffrey Karnes, Michael Koch, Courtney Rose Dhondt, Joel B. Nelson, Dawn McBride, Michael S. Cookson, Kelly L. Stratton, Stephen Farriester, Erin Hemken, Walter M. Stadler, Tuula Pera, Deimante Banionyte, Fernando J. Bianco Jr., Isabel H. Lopez, Stacy Loeb, Samir S. Taneja, Nataliya Byrne, Christopher L. Amling, Ann Martinez, Luc Boileau, Franklin D. Gaylis, Jacqueline Petkewicz, Nicholas Kirwen, Brian T. Helfand, Jianfeng Xu, Denise M. Scholtens, William J. Catalona, and John S. Witte

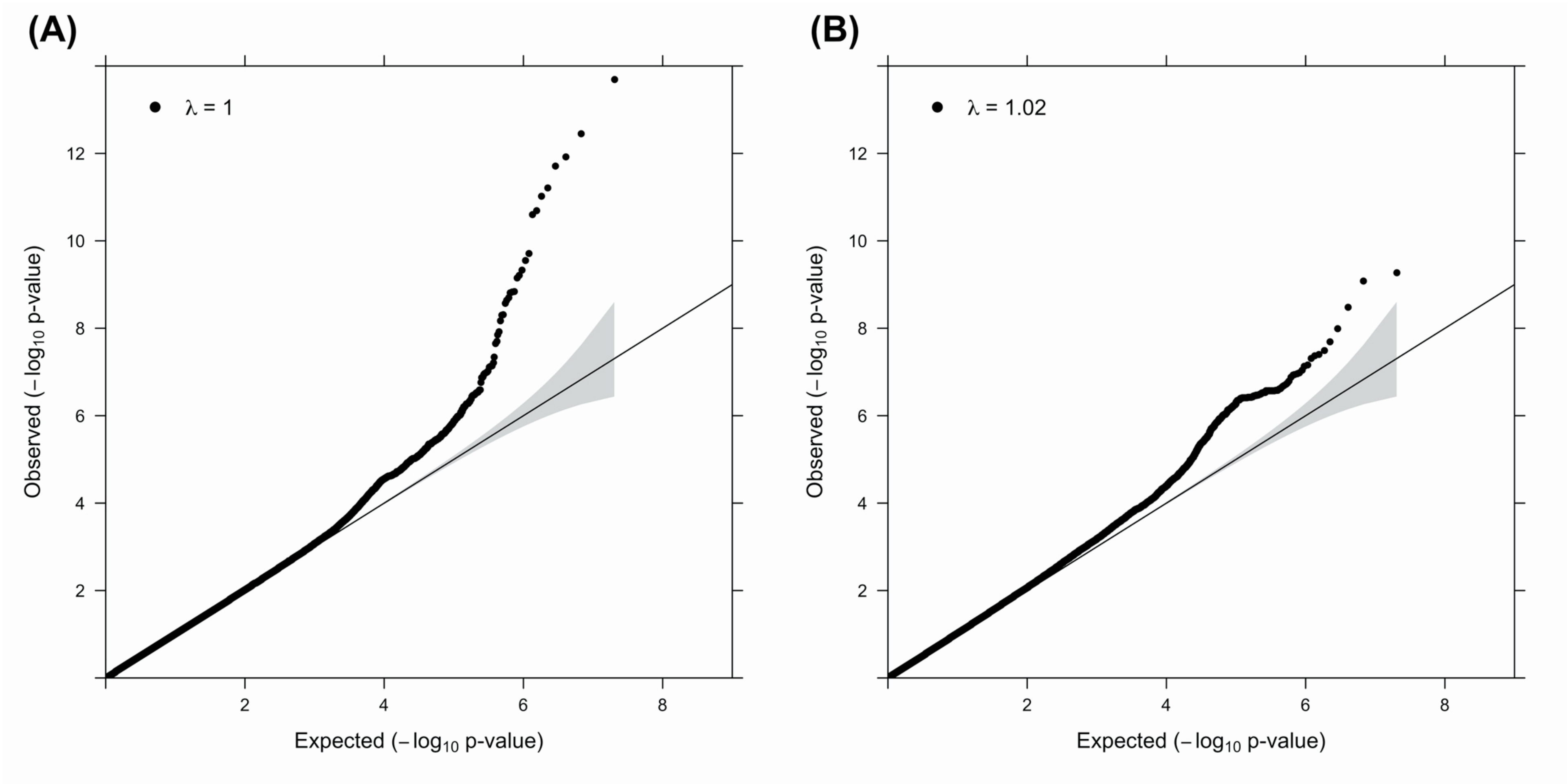

Figure S1. QQ plots of the GWASs. (A) QQ plot of GWAS in individuals of European ancestry. (B) QQ plot of combined GWAS meta-analysis of all individuals. Genomic control λ is shown.

**Discovery Samples**		
Restriction	N Excluded	Total N
Genotyped at CIDR		6,324
independent individuals	23	6,301
With > 6 months follow-up or no missing follow-up	344	5957
With no missing age/censoring status	21	5936
Genetically European	714	5,222
Final N in discovery samples		5,222

**Replication Samples**		
Restriction	N Excluded	Total N
Genotyped at MD Anderson		593
With >6 months follow-up or no missing follow-up	72	591
Non-censored	37	484
Genetically European	59	425
Non-European from CIDR		714
Final N in replication samples		1,139

Table S1: Summary of subjects included and excluded from the discovery GWAS and replication for the study of active surveillance conversion to treatment.

	Discovery		Replication							
Ancestral population	European		African		Asian		Latin American		European[a]	
Sample size	5,222		396		237		81		425	
Mean age (± s.d.)	63.5	(±7.2)	62	(±8.2)	63.8	(±7.7)	63	(±7.2)	64.4	(±8.2)
Median follow-up time (months, IQR)	80	(51,112)	68	(40,106)	86	(54,118)	59	(33,85)	80	(31,110)
Gleason grade										
GG1	4,819	(92%)	359	(91%)	219	(92%)	77	(95%)	372	(87%)
GG2	344	(7%)	32	(8%)	15	(6%)	4	(5%)	48	(11%)
≥ GG3	55	(1%)	4	(1%)	3	(1%)	0	(0%)	5	(1%)
PSA (MAD)	5.0	(±2.1)	5.3	(±2.0)	5.3	(±2.5)	4.7	(±2.4)	4.1	(±2.2)
Clinical tumor stage										
cT1	4,138	79%	322	81%	161	68%	66	81%	372	88%
cT2	641	12%	49	12%	39	16%	9	11%	53	12%
cT3 or cT4	34	1%	5	1%	5	2%	1	1%	0	0%
Number of cores										
1-2	4,113	(79%)	298	(75%)	177	(75%)	67	(83%)	386	(91%)
3	451	(9%)	44	(11%)	20	(8%)	7	(9%)	27	(6%)
≥ 4	522	(10%)	39	(10%)	37	(16%)	7	(9%)	12	(3%)
PC risk category										
Low	3,639	(70%)	262	(66%)	141	(59%)	59	(73%)	314	(74%)
Intermediate	983	(19%)	82	(21%)	53	(22%)	14	(17%)	91	(21%)
High	599	(11%)	52	(13%)	43	(18%)	8	(10%)	20	(5%)
Number of conversions	1609		123		88		21		77	
Tumor upgrading	920	(57%)	68	(55%)	51	(58%)	13	(62%)	44	(57%)
Tumor volume progression	147	(9%)	10	(8%)	6	(7%)	0	(0%)	53	(69%)

PSA Progression	219	(14%)	20	(16%)	15	(17%)	0	(0%)	3	(4%)
Other Reason	134	(8%)	14	(11%)	4	(5%)	2	(10%)	4	(5%)
No Reason Reported	317	(20%)	21	(17%)	20	(23%)	6	(29%)	9	(12%)

Table S2. Characteristics of active surveillance patients included in the discovery and replication genome-wide association study.
a. MD Anderson samples of European Ancestry.
IQR, interquartile range; MAD, median absolute deviation; PSA, prostate-specific antigen; PC, prostate cancer.
Men of genetically inferred European ancestry genotyped by CIDR are included in the discovery GWAS. The other participants genotyped by CIDR and men from MD Anderson are included in the replication.
Age and cancer clinical characteristics were measured at diagnosis.
PC risk categories:

Low-risk patients had all the following criteria: GG1 only (Gleason ≤ 3+3), PSA <10 ng/mL, clinical stage T1, and ≤ 3 positive biopsy cores.

Intermediate-risk patients had any of the following, with no high-risk criteria: GG2 (Gleason 3+4), PSA 10-20 ng/mL, or clinical stage T2.

High-risk patients had any of the following: ≥ GG3 (≥ Gleason 4+3), PSA ≥20 ng/mL, clinical stage ≥T3, or ≥ 4 positive biopsy cores.

Percentages do not all sum to 100% due to missingness.

Model	GRS_{PC}[d]			GRS_{PSA}[d]			AUC
	HR	95% CI	P-value	HR	95% CI	P-value	
Reference model[a]							0.550
PC clinical characteristics[b]							0.653
PC GRS[c]	1.18[e]	1.12, 1.23	5.6×10^{-11}				0.576
PSA GRS[c]				0.92[e]	0.88, 0.96	3.9×10^{-4}	0.557
PC clinical characteristics + PC GRS[c]	1.13[e]	1.07, 1.19	3.3×10^{-6}				0.659
PC clinical characteristics + PSA GRS[c]				0.94[e]	0.89, 0.98	8.5×10^{-3}	0.655
PC clinical characteristics + PC GRS + PSA GRS	1.15[e]	1.09, 1.22	1.5×10^{-7}	0.91[e]	0.87, 0.96	3.0×10^{-4}	0.661

Table S3. **ROC analysis of PC and PSA GRS compared to clinical characteristics for conversion from AS to treatment.**

[a] All models contain age and the first 10 principal components.

[b] PC clinical characteristics were Gleason grade group (GG1, GG2, or ≥ GG3); PSA concentration (ng/mL); clinical stage (cT1, cT2, or cT3/cT4); and number of positive biopsy cores (1-2, 3, or ≥ 4).

[c] PC GRS is constructed from 269 prostate cancer associated variants, while PSA GRS is derived from 36 PSA risk variants.

[d] The test statistics of GRS_{PC} and GRS_{PSA} are reported when the model contains them.

[e]The HRs correspond to a one SD increase in the GRS.

	Minimally Adjusted			Fully Adjusted		
	HR[a]	95% CI	P	HR[b]	95% CI	P
**Decile**						
0-10	0.73	0.60, 0.89	0.0016	0.69	0.56, 0.86	0.0010
10-20	0.81	0.68, 0.98	0.030	0.83	0.68, 1.01	0.067
20-30	0.93	0.78, 1.11	0.44	0.99	0.82, 1.20	0.95
30-40	0.83	0.69, 0.99	0.041	0.82	0.67, 0.99	0.043
40-60	1.00	Reference		1.00	Reference	
60-70	1.06	0.89, 1.26	0.50	1.03	0.86, 1.24	0.72
70-80	1.10	0.92, 1.30	0.30	1.02	0.85, 1.22	0.87
80-90	1.23	1.04, 1.46	0.014	1.15	0.96, 1.37	0.13
90-100	1.27	1.07, 1.51	0.0061	1.13	0.94, 1.36	0.18

Table S4. Hazard ratios for the association between time to AS failure with prostate cancer GRS.
HR = Hazard Ratio; CI = Confidence Interval

[a] Hazard ratios are adjusted for age and the first 10 genetic principal components

[b] Hazard ratios are adjusted for age, the first 10 genetic principal components, Gleason grade group (GG1, GG2, or ≥ GG3); PSA concentration (ng/mL); clinical stage (cT1, cT2, or cT3/cT4); and number of positive biopsy cores (1-2, 3, or ≥ 4).

	Minimally Adjusted			Fully Adjusted		
	HR[a]	95% CI	P	HR[b]	95% CI	P
**Decile**						
0-10	1.28	1.08, 1.52	0.0044	1.25	1.04, 1.50	0.017
10-20	1.08	0.90, 1.29	0.40	1.01	0.83, 1.22	0.96
20-30	1.15	0.97, 1.37	0.11	1.18	0.98, 1.42	0.083
30-40	1.16	0.98, 1.38	0.088	1.16	0.96, 1.39	0.12
40-60	1.00	Reference		1.00	Reference	
60-70	1.08	0.90, 1.29	0.40	1.05	0.86, 1.27	0.65
70-80	1.00	0.83, 1.19	0.96	0.96	0.79, 1.17	0.70
80-90	0.98	0.82, 1.18	0.84	1.02	0.84, 1.24	0.87
90-100	0.95	0.79, 1.15	0.60	0.97	0.79, 1.19	0.78

Table S5. Hazard ratios for the association between time to AS failure with prostate-specific antigen GRS.

HR = Hazard Ratio; CI = Confidence Interval

[a] Hazard ratios are adjusted for age and the first 10 genetic principal components

[b] Hazard ratios are adjusted for age, the first 10 genetic principal components, Gleason grade group (GG1, GG2, or ≥ GG3); PSA concentration (ng/mL); clinical stage (cT1, cT2, or cT3/cT4); and number of positive biopsy cores (1-2, 3, or ≥ 4).
