# Supplementary material for: Genetic factors associated with prostate cancer conversion from active surveillance to treatment
Source: HGG Adv. 2021 Nov 19;3(1):100070. doi: 10.1016/j.xhgg.2021.100070 (PMC8725988; doi:10.1016/j.xhgg.2021.100070)
Supplement: Document S2. Article plus supplemental information [file mmc2.pdf]

HGG Advances

ARTICLE

Genetic factors associated with prostate cancer conversion from active surveillance to treatment

Yu Jiang,[1,41] Travis J. Meyers,[1,41] Adaeze A. Emeka,[2] Lauren Folgosa Cooley,[2] Phillip R. Cooper,[2] Nicola Lancki,[3] Irene Helenowski,[3] Linda Kachuri,[1] Daniel W. Lin,[4,5] Janet L. Stanford,[6,7] Lisa F. Newcomb,[4,5] Suzanne Kolb,[6,7] Antonio Finelli,[8] Neil E. Fleshner,[8] Maria Komisarenko,[8] James A. Eastham,[9] Behfar Ehdaie,[9] Nicole Benfante,[9] Christopher J. Logothetis,[10] Justin R. Gregg,[10] Cherie A. Perez,[10] Sergio Garza,[10] Jeri Kim,[10] Leonard S. Marks,[11] Merdie Delfin,[11] Danielle Barsa,[11] Danny Vesprini,[12] Laurence H. Klotz,[12] Andrew Loblaw,[12] Alexandre Mamedov,[12] S. Larry Goldenberg,[13] Celestia S. Higano,[13] Maria Spillane,[13] Eugenia Wu,[13] H. Ballentine Carter,[14] Christian P. Pavlovich,[14] Mufaddal Mamawala,[14] Tricia Landis,[14] Peter R. Carroll,[15] June M. Chan,[1,15] Matthew R. Cooperberg,[15,16] Janet E. Cowan,[15] Todd M. Morgan,[17] Javed Siddiqui,[18] Rabia Martin,[18] Eric A. Klein,[19] Karen Brittain,[19] Paige Gotwald,[19] Daniel A. Barocas,[20]

(Author list continued on next page)

Summary

Men diagnosed with low-risk prostate cancer (PC) are increasingly electing active surveillance (AS) as their initial management strategy. While this may reduce the side effects of treatment for PC, many men on AS eventually convert to active treatment. PC is one of the most heritable cancers, and genetic factors that predispose to aggressive tumors may help distinguish men who are more likely to discontinue AS. To investigate this, we undertook a multi-institutional genome-wide association study (GWAS) of 5,222 PC patients and 1,139 other patients from replication cohorts, all of whom initially elected AS and were followed over time for the potential outcome of conversion from AS to active treatment. In the GWAS we detected 18 variants associated with conversion, 15 of which were not previously associated with PC risk. With a transcriptome-wide association study (TWAS), we found two genes associated with conversion (*MAST3*, $p = 6.9 \times 10^{-7}$ and *GAB2*, $p = 2.0 \times 10^{-6}$). Moreover, increasing values of a previously validated 269-variant genetic risk score (GRS) for PC was positively associated with conversion (e.g., comparing the highest to the two middle deciles gave a hazard ratio [HR] = 1.13; 95% confidence interval [CI] = 0.94–1.36); whereas decreasing values of a 36-variant GRS for prostate-specific antigen (PSA) levels were positively associated with conversion (e.g., comparing the lowest to the two middle deciles gave a HR = 1.25; 95% CI, 1.04–1.50). These results suggest that germline genetics may help inform and individualize the decision of AS—or the intensity of monitoring on AS—versus treatment for the initial management of patients with low-risk PC.

Introduction

Active surveillance (AS) is now more widely implemented as an initial management strategy for many men with lower-risk prostate cancer (PC [MIM: 176807]).[1] PC that is unlikely to invade surrounding tissue or metastasize according to characteristics at diagnosis is considered low-risk or favorable-intermediate risk.[2] Recent work in the United States Veterans Administration (VA) Health Care System[3,4] and in Sweden[5] indicates that a majority of men with low-risk PC are being managed with AS. Determining which patients most benefit from early active treatment versus AS, however, and how intensive the surveillance protocol should be, remains a challenge.

A major drawback of AS for low-risk PC is the possibility of misclassifying patients with a life-threatening disease. In fact, over a 10-year follow-up period, 20%–40% of men initially managed with AS later have more aggressive cancer.[6] While the impact of delayed treatment is unknown, up to 50% of men in one AS series of studies experienced

[1]Department of Epidemiology and Biostatistics, University of California, San Francisco, San Francisco, CA 94158, USA; [2]Department of Urology, Northwestern University Feinberg School of Medicine, Chicago, IL 60611, USA; [3]Division of Biostatistics, Department of Preventive Medicine, Northwestern University Feinberg School of Medicine, Chicago, IL 60611, USA; [4]Fred Hutchinson Cancer Research Center, Cancer Prevention Program, Public Health Sciences, Seattle, WA 98109, USA; [5]Department of Urology, University of Washington, Seattle, WA 98195, USA; [6]Fred Hutchinson Cancer Research Center, Cancer Epidemiology Program, Public Health Sciences, Seattle, WA 98109, USA; [7]Department of Epidemiology, University of Washington, School of Public Health, Seattle, WA 98195, USA; [8]Division of Urology, Department of Surgery, Princess Margaret Cancer Centre, University Health Network, Toronto, ON, Canada; [9]Urology Service, Department of Surgery, Memorial Sloan Kettering Cancer Center, New York, NY, USA; [10]Departments of Genitourinary Medical Oncology and Urology, University of Texas MD Anderson Cancer Center, Houston, TX, USA; [11]Department of Urology, David Geffen School of Medicine at UCLA, Los Angeles, CA, USA; [12]Odette Cancer Centre, Sunnybrook Health and Sciences Centre, University of Toronto, Toronto, ON, Canada; [13]Department of Urologic Sciences, University of British Columbia, Vancouver, BC, Canada; [14]Brady Urological Institute, Johns Hopkins University School of Medicine, Baltimore, MD, USA; [15]Department of Urology, University of California, San Francisco, San Francisco, CA, USA; [16]Helen Diller Family Comprehensive

(Affiliations continued on next page)

© 2021 The Author(s). This is an open access article under the CC BY license (http://creativecommons.org/licenses/by/4.0/).

Jeremiah R. Dallmer,[20,21] Jennifer B. Gordetsky,[20,22] Pam Steele,[20] Shilajit D. Kundu,[2] Jazmine Stockdale,[2] Monique J. Roobol,[23] Lionne D.F. Venderbos,[23] Martin G. Sanda,[24] Rebecca Arnold,[24] Dattatraya Patil,[24] Christopher P. Evans,[25] Marc A. Dall'Era,[25] Anjali Vij,[25] Anthony J. Costello,[26] Ken Chow,[26] Niall M. Corcoran,[26] Soroush Rais-Bahrami,[27,28] Courtney Phares,[27] Douglas S. Scherr,[29] Thomas Flynn,[29] R. Jeffrey Karnes,[30] Michael Koch,[31] Courtney Rose Dhondt,[31] Joel B. Nelson,[32] Dawn McBride,[32] Michael S. Cookson,[33] Kelly L. Stratton,[33] Stephen Farriester,[33] Erin Hemken,[33] Walter M. Stadler,[34] Tuula Pera,[34] Deimante Banionyte,[34] Fernando J. Bianco, Jr.,[35] Isabel H. Lopez,[35] Stacy Loeb,[36] Samir S. Taneja,[36] Nataliya Byrne,[36] Christopher L. Amling,[37] Ann Martinez,[37] Luc Boileau,[37] Franklin D. Gaylis,[38] Jacqueline Petkewicz,[39] Nicholas Kirwen,[39] Brian T. Helfand,[39] Jianfeng Xu,[39] Denise M. Scholtens,[3] William J. Catalona,[2,42] and John S. Witte[1,15,16,40,42,*]

biochemical recurrence after active treatment.[7] These uncertainties and challenges in accurately discriminating between indolent and aggressive PC may prompt men to err on the side of early treatment, resulting in unnecessary side effects and worse health-related quality of life, or conversely result in delays in therapy for men who are likely to benefit from it. Many men have such low-risk disease that they do not need the biopsies or scans with the frequency with which they have typically been performed. Recent work suggests that it might be possible to predict the likelihood of risk reclassification of an affected individual's disease (for at least 4 years of AS[8]). Thus, it may be possible to reduce the intensity of surveillance for many men with the lowest-risk tumors.

A key outstanding question is how to best distinguish among low- and high-risk tumors for AS decisions. Promising recent developments for enhancing clinical risk assessment include multi-parametric magnetic resonance imaging (MRI) with targeted prostate biopsy and tissue-based genomic testing.[9,10] Another potentially valuable approach is incorporating germline genetic information for PC via a polygenic risk score.[11] PC is one of the most heritable of common cancers, with germline genetic factors accounting for over 40% of the variability in this disease.[12–15] We and others have identified from genome-wide association studies (GWASs) 269 common germline genetic variants associated with PC susceptibility that explain a substantial proportion of disease heritability.[16–43] Combining these PC risk variants into a genetic risk score (GRS) may provide a more discriminatory biomarker not only for PC risk but also potentially for predicting conversion from AS to treatment.[44–47] Moreover, we recently have discovered genetic variants that explain variability in prostate-specific antigen (PSA) levels.[48] Since PSA is a critical component to monitoring men undergoing AS, incorporating this information may also help to identify ideal AS candidates.

To evaluate the potential value of incorporating germline genetic information into the shared decision-making process for AS, we present findings from a large, multi-institutional GWAS of men diagnosed with PC enrolled in an AS program. We report novel variants and genes and GRSs associated with conversion from AS to treatment.

Material and methods

Participants

The primary study participants came from 28 institutions in the United States, Canada, the Netherlands, and Australia. We recently reported on the clinicopathological characteristics of conversion to treatment in this population.[49] The AS cohort genotyped by the Center for Inherited Disease Research (CIDR) included 6,324 men diagnosed with PC between 1991 and 2018 who elected AS for their initial management. We also included an additional 593 AS patients from the University of Texas MD Anderson Cancer Center as replication samples, described below. Patients' blood or tissue samples were collected to conduct germline

Cancer Center, University of California, San Francisco, San Francisco, CA, USA; [17]Department of Urology, University of Michigan, Ann Arbor, MI, USA; [18]Department of Pathology, University of Michigan, Ann Arbor, MI, USA; [19]Glickman Urological and Kidney Institute, Cleveland Clinic Lerner College of Medicine, Cleveland Clinic, Cleveland, OH, USA; [20]Department of Urology, Vanderbilt University Medical Center, Nashville, TN, USA; [21]Department of Urology, Cedars-Sinai Medical Center, Los Angeles, CA, USA; [22]Department of Pathology, Microbiology, and Immunology, Vanderbilt University Medical Center, Nashville, TN, USA; [23]Department of Urology, Erasmus Cancer Institute, Erasmus University Medical Center, Rotterdam, the Netherlands; [24]Department of Urology, Emory University School of Medicine, Atlanta, GA, USA; [25]Department of Urologic Surgery, University of California, Davis Medical Center, Sacramento, CA, USA; [26]Department of Urology, Royal Melbourne Hospital and University of Melbourne, Melbourne, VIC, Australia; [27]Department of Urology, University of Alabama at Birmingham, Birmingham, AL, USA; [28]Department of Radiology, University of Alabama at Birmingham, Birmingham, AL, USA; [29]Department of Urology, Weill Cornell Medicine, New York-Presbyterian Hospital, New York, NY, USA; [30]Mayo Clinic Department of Urology, Rochester, MN, USA; [31]Department of Urology, Indiana University School of Medicine, Indianapolis, IN, USA; [32]Department of Urology, University of Pittsburgh School of Medicine, Pittsburgh, PA, USA; [33]Department of Urology, University of Oklahoma Health Sciences Center, Oklahoma City, OK, USA; [34]University of Chicago Comprehensive Cancer Center, Chicago, IL, USA; [35]Urological Research Network, Miami Lakes, FL, USA; [36]Departments of Urology and Population Health, New York University Langone Health and Manhattan Veterans Affairs Medical Center, New York, NY, USA; [37]Department of Urology, Oregon Health and Science University, Portland, OR, USA; [38]Genesis Healthcare Partners, Department of Urology, University of California, San Diego, CA, USA; [39]Division of Urology, NorthShore University Health System, Evanston, IL, USA; [40]Departments of Epidemiology and Population Health, Biomedical Data Science, and Genetics, Stanford University, Stanford, CA, USA
[41]These authors contributed equally
[42]These authors contributed equally
*Correspondence: jswitte@stanford.edu
https://doi.org/10.1016/j.xhgg.2021.100070.

genetic analyses. The AS protocols varied among participating institutions, reflecting real-world practice patterns,[50–53] and we did not impose strict inclusion/exclusion criteria based on the AS protocol. Patient demographic and clinical variables were collected and managed using the Research Electronic Data Capture (REDCap) software.[54,55] Among the samples genotyped at CIDR, individuals were excluded from further analyses for the following reasons (Table S1): (1) being related to another participant at the 3rd degree or closer (n = 23; 0.36%), (2) unknown or <6 months on AS (n = 344; 5.4%), or (3) missing information on age and censoring status (n = 21; 0.33%). This left us with 5,936 men from the CIDR genotyping for inclusion in the discovery GWAS (n = 5,222) and the non-European (non-EUR) replication GWAS (n = 714). This study was approved by the institutional review board at each institution, all participants provided written informed consent, and all participating institutions signed a material transfer and data use agreement.

Clinical and demographic factors

We collected PC characteristics at diagnosis, including the age at diagnosis, Gleason grade group (GG), PSA level, clinical tumor stage (cT), and the number of cancerous biopsy cores at diagnosis. Grade groups correspond to the following Gleason scores (GSs): GG1, ~GS ≤ 6; GG2, ~GS 3+4; GG3, ~GS 4+3; GG4, ~GS 8; GG5, ~GS 9 or 10.[56] Study participants were classified into three risk groups (low, intermediate, and high risk) based on our modification of guidelines from the National Comprehensive Cancer Network (NCCN) and the American Urological Association (AUA). We did not strictly follow these guidelines because we were unable to distinguish between cT2a, cT2b, and cT2c, and we did not have data on PSA density (serum PSA concentration divided by prostate volume). Therefore, low-risk patients met the following criteria: GG1 (GS 3+3), PSA < 10 ng/mL, cT1, and ≤3 positive biopsy cores. Intermediate-risk patients had any of the following without any high-risk or high-volume criteria: GG2 (GS 3+4), PSA 10–20 ng/mL, or stage cT2. High-risk patients had any of the following: ≥GG3 (≥GS 4+3), PSA ≥ 20 ng/mL, stage ≥ cT3, or ≥4 positive biopsy cores of any GG.

Conversion occurred when an affected individual received treatment following AS. The reason for withdrawing from AS to begin treatment was reported as due to "upgrading," "upstaging," "PSA progression," "anxiety," and/or "other" reasons. Note that in our survival analysis (below), individuals who converted due to anxiety were censored and do not contribute events in our analysis. We used the ADMIXTURE software program to infer genetic ancestry from uncorrelated variants, according to major reference populations in the 1000 Genomes Project (European, African, East/South Asian combined, and Admixed American).[57]

Genotyping and imputation

In total, 6,324 participants were genotyped on the Illumina Infinium Multi-Ethnic Global Array (MEGA), including custom content, at the NIH CIDR at Johns Hopkins University. Genotypes were called using GenomeStudio version 2011.1, genotyping module version 1.9.4, and GenTrain version 1.0. The full array with custom content consisted of 1,760,143 variants. For 99 of our study subjects, DNA was obtained from normal seminal vesicle tissue, which is an accurate source of germline genetic variants.[58] Even if some of the variants in these individuals are somatic, this should not impact our results, since <2% of the study population had DNA obtained from seminal vesicle tissue.

After genotyping, the median variant call rate was 99.94%, and the error rate estimated from 122 pairs of planned study duplicates was 1.3×10^{-6}. Samples and variants were excluded if they had a sample or genotyping call rate < 98%. We limited our analyses to variants with a minor allele frequency (MAF) ≥ 1%. Variants were screened for deviations from Hardy-Weinberg equilibrium with a filter threshold of $p = 6.5 \times 10^{-4}$. A total of 856,077 genotyped variants remained after these quality control (QC) steps. Unmeasured genetic variants were imputed using the Trans-Omics for Precision Medicine (TOPMed) imputation server, with 97,256 reference samples and 308,107,085 variants. Variants with imputation quality (INFO) score < 0.3 were excluded, leaving a total of 22,691,641 variants successfully imputed. The QC steps checked for related individuals using the R package SNPRelate. We found that 23 pairs were related at third-degree relative or closer and retained from each of these pairs the individual with the longest follow-up. After QC steps, a total of 5,936 samples genotyped at CIDR remained for inclusion in the GWAS. Eighty-eight percent of these men (5,222) were inferred as genetically European (using the program ADMIXTURE) and comprised the discovery cohort. The remaining 714 non-European men were included in our replication analysis. In this study, we use only genetic ancestry.

Furthermore, we included in our analysis an additional 593 AS patients from MD Anderson previously genotyped on the Illumina Infinium OncoArray-500K BeadChip Array. This array was primarily developed to study cancer predisposition and risk. Genotypes were called using GenomeStudio version 2011.1. The full array consisted of 500,000 variants. Genotype QC procedures and imputation for the PRACTICAL OncoArray have been described previously.[15] Briefly, imputation was performed without prephasing with SHAPEIT2 based on the 1000 Genomes phase 3 release reference panel. In total, 21,299,194 variants were successfully imputed, and 10,109,977 variants with MAF ≥ 1% on autosomal chromosomes 1–22 and sex chromosome X. The MD Anderson AS samples included a total of 593 men. Of these, we excluded the following (Table S1): (1) men without data on the duration of AS or those managed with AS for less than six months (n = 72; 12%), (2) those without data on censoring status (n = 37; 6.2%), and (3) those genetically non-European (n = 59; 9.9%). This left us with 425 men from MD Anderson for replication (in addition to the non-European men genotyped at CIDR).

GWAS of conversion from AS to treatment

The variants with MAF ≥ 1% on autosomal chromosomes 1–22 and sex chromosome X were tested for their association with time to conversion from AS to treatment among the 5,222 men of European genetic ancestry genotyped by CIDR. Patients who converted due to anxiety were censored because the event of interest was converting due to a change in the cancer clinical characteristics. Per-allele hazard ratios (HRs), 95% confidence intervals (CIs), and corresponding p values were calculated from Cox proportional hazards models. HRs were adjusted for age at diagnosis and the first 10 genetic principal components to address potential population stratification or cryptic relatedness. Adjusted HRs were calculated using the gwasurvivr package in R.[59] For any variants associated with conversion, we examined the Cox models' proportional hazards assumption.

Following the GWAS discovery phase, the potential associations were tested for replication in an independent GWAS among 714 men also genotyped by CIDR (but of non-European ancestry)

and the 425 MD Anderson samples of European genetic ancestry (excluding other ancestries), adjusting for age and 10 principal components. Again, variants with MAF $\geq$ 1% on autosomal chromosomes and sex chromosome X were tested for their association with conversion within major ancestral populations (i.e., European, African, Asian, and Admixed American). For the MD Anderson patients, 9,962,324 variants were tested in a Cox proportional hazards model adjusted for age at PC diagnosis and ancestry principal components.

Results from the GWAS were combined with a fixed-effects inverse-variance-weighted meta-analysis using METAL.[60] All statistical tests were two-sided. Marginal p values less than 5×10^{-8} were considered statistically significant. We defined a locus as the 1 Mb region surrounding the sentinel variant (500 kb pairs flanking each side). To identify independently associated variants, within each 1 Mb region we performed clumping on the association results using PLINK v.1.9 using a linkage disequilibrium threshold $r^2 < 0.5$). We assess and report heterogeneity for the top variants in the meta-analysis of discovery study and replication meta-analysis.

Transcriptome-wide association study of conversion from AS to treatment

To identify additional genes associated with time to conversion, we conducted a transcriptome-wide association analysis (TWAS), which models genetically imputed transcript levels and has a lower multiple testing burden compared to single-variant analysis. We applied the MetaXcan analytic pipeline to our combined GWAS summary statistics and associated genetically predicted expression of approximately 22,000 genes across a 49-issue reference dataset from GTEx (version 8).[61] Tissue-specific associations were aggregated using S-MultiXcan to obtain cross-tissue p values for each gene.[61] Associations were considered statistically significant at the Bonferroni-corrected alpha level of 2.2×10^{-6} (i.e., 0.05/22,535 genes).

GRSs

GRSs were constructed by summing variant-specific weighted allelic dosages for the samples genotyped by CIDR. The initial GRS included the 269 PC risk variants reported in the largest trans-ancestry GWAS meta-analysis of PC.[43] Specifically, for patient *i*, $GRS_i = \sum_{m=1}^{M} w_m g_{im}$, where g_{im} is the genotype dosage for patient *i*, and variant *m*, and w_m is the variant weight on the log odds ratio scale from the published literature (i.e., the meta-analysis for the GRS_{PC}). *M* is the total number of variants included in the GRS ($M = 269$ for the GRS_{PC}). A second GRS was developed for the genetic basis of serum PSA levels. This GRS_{PSA} included 36 variants and their corresponding weights from a GWAS of PSA levels among cancer-free men.[48] First, the associations between these GRSs and conversion were estimated using multivariable Cox proportional hazards models, where GRS is a continuous variable. Minimally adjusted Cox models included continuous GRS, age, and the first 10 genetic principal components. Fully adjusted Cox models also included Gleason grade group (GG1, GG2, or $\geq$ GG3), PSA concentration (ng/mL), clinical stage (cT1, cT2, or cT3/cT4), and the number of positive biopsy cores (1–2, 3, or $\geq$4). We adjust for those additional factors because they are independently associated with AS outcomes. Including them in the fully adjusted models allows us to assess whether the PC GRS provides additional information in predicting AS treatment conversion. In addition, by undertaking these additional fully adjusted analyses, we can assess how sensitive our GRS results from the minimally adjusted models are to including known clinical factors. Second, we next assessed whether the GRS allowed risk stratification of conversion from AS to active treatment. The GRS was modeled as a categorical variable according to deciles of the distribution. We calculated the GRS decile cutoffs based on the full study population. Essentially identical cutoffs and results were observed when basing the cutoffs on men who did not end AS. HRs were estimated for each GRS decile relative to the average 40%–60% category using a minimally and a fully adjusted Cox model. Individuals with missing variables were removed from the GRS analyses.

Clinical utility of the GRS

The potential utility of the GRS was evaluated by comparing how the top and bottom deciles of the GRS distribution modified conversion rates within the three PC clinicopathological risk categories (i.e., low, intermediate, and high). For the top and bottom GRS deciles (top 10th percentile and bottom 10th percentile, respectively) we plotted Kaplan-Meier curves of conversion within each PC clinicopathological risk category and tested the difference between each pair of curves with the log-rank test.

To evaluate the overall discriminative capacity of the GRS (i.e., not just the decile tails), we calculated the area under the receiver operating characteristic (ROC) curve (AUC) in the discovery sample using regression models of time to conversion. We used Chambless and Diao's estimator of cumulative AUC for right-censored time-to-event data, which is a summary measure given by the integral of AUC on [0, max(times)] weighted by the estimated probability density of the time-to-event outcome.[62] A baseline AUC was calculated for the model that included age and the first 10 principal components. This model was then expanded to further include PC clinical characteristics listed above for the multivariable Cox model, followed by GRS_{PC} and GRS_{PSA} (individually and together).

Ethical publication statement

This study was approved by the Robert H. Lurie Comprehensive Cancer Center of Northwestern University Scientific Review (IRB) committees. The approval number is STU00077147, which was most recently given annual approval on July 8, 2021.

Results

Study population

Table S1 provides details of sample inclusions and exclusions. Of the 1,659 patients who converted from AS to treatment, 50 patients in the discovery sample reported anxiety as one of the reasons for conversion and were censored and did not contribute events in our analysis. Over half of the patients who converted reported tumor grade reclassification as one of the reasons for conversion for both discovery (920/1,609; Table S2) and replication samples (176/309; Table S2). Details of the discovery and replication samples that met inclusion criteria are presented in Table S2. The study characteristics in Table S2 were defined at PC diagnosis. Clinically, most men in the discovery sample had low-risk PC (3,639; 70%) and/or

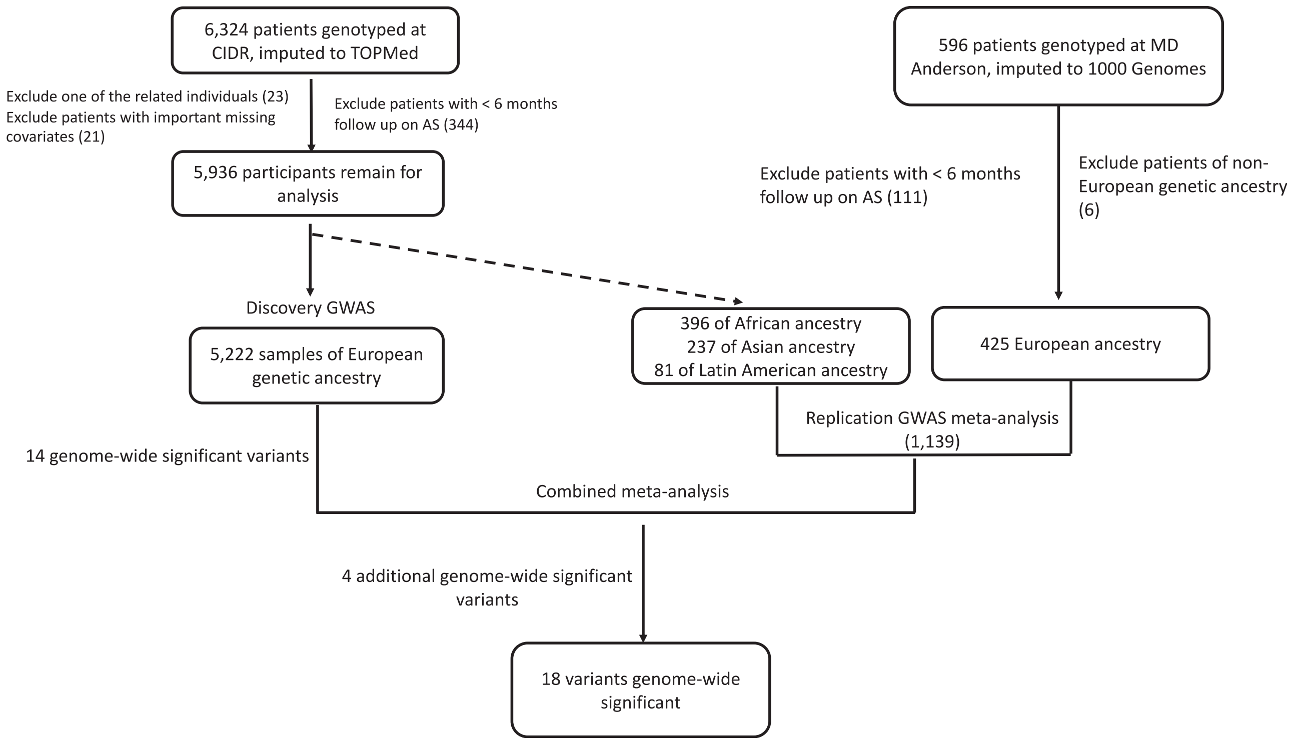

Figure 1. Flow chart highlighting the approach and samples used in the genome-wide association analysis
First, we undertook a discovery GWAS in men of European ancestry. Fourteen variants were associated with conversion ($p < 5 \times 10^{-8}$). All variants were evaluated for replication in the replication cohorts alone and then in a meta-analysis combining the discovery and replication cohorts. Four additional variants reached statistical significance in the combined meta-analysis ($p < 5 \times 10^{-8}$).

features of low-risk, low-volume disease: GG1 (4,819; 92%), 1–2 positive biopsy cores (4,113; 79%), and a median PSA at diagnosis of 5 ng/mL. The demographic and clinicopathological characteristics of the replication samples had a similar pattern as the discovery samples, except that the proportion of high-risk PC was higher for men of Asian genetic ancestry (n = 43; 18%) than of European ancestry (n = 599; 11%; Table S2). Baseline characteristics were missing for the following proportion of study participants: age at diagnosis (<0.1%), GG group (<0.1%), PSA concentration (3.3%), clinical tumor stage (6.9%), number of positive biopsy cores (2.5%), and risk-group classification (<0.1%).

GWAS of conversion from AS to treatment

Our approach to the GWAS discovery, replication, and meta-analysis is outlined in Figure 1. The median follow-up time for patients in this multicenter study was 6.7 years. Our primary discovery GWAS yielded 14 independent lead variants (i.e., p value $< 5 \times 10^{-8}$ at each locus of size 1 Mb) (Figure 2A). We replicated 1 of the signals at a p value level less than 0.05/14 (≈0.0036) in the replication meta-analysis. In the combined meta-analysis of discovery and replication GWASs, we detected four additional variants independently associated with conversion to treatment (Figure 2B). We have undertaken tests for heterogeneity and indicated those with $I^2 > 0.75$ (Table 1); the inconsistency in these results suggests that they may be unstable results that merit further replication. Q-Q plots for the discovery GWAS and the combined meta-analysis did not suggest inflation of test statistics due to systematic bias such as population substructure (genomic inflation factor = 1 and 1.02, respectively; Figure S1).

Of the 18 variants, four were common (MAF > 0.01) and the remainder were low frequency (MAF = 0.01) (Table 1). Three were located within 1 Mb of previous PC GWAS-identified variants, although they were not in linkage disequilibrium with these variants ($r^2 < 0.3$). These were: intronic variant rs4721243 of *MAD1L1* (MIM: 602686) on chromosome 7 (CIDR European GWAS HR = 5.65, p = 7×10^{-10}); rs1404610 near *GLI2* (MIM: 165230; combined meta-analysis HR = 3.74, p = 5.4×10^{-10}) on chromosome 2; and rs74874116 near *GATA5* (MIM: 611496; CIDR European HR = 2.67, p = 1.47×10^{-9}) on chromosome 20.

TWAS of conversion from AS to treatment

In the multi-tissue TWAS analysis using S-MultiXcan, the imputed expression levels of two genes were associated with conversion after Bonferroni correction for multiple testing: *MAST3* (MIM: 612258; p value = 6.9×10^{-7}) and *GAB2* (MIM: 606203; p value = 2.0×10^{-6}). Imputed expression levels of two other genes suggested an association with conversion: *ARRDC2* (p value = 2.7×10^{-5}) and *CELSR1* (MIM: 604523; p value = 9.5×10^{-5}). When looking only at prostate tissue, we observed modest

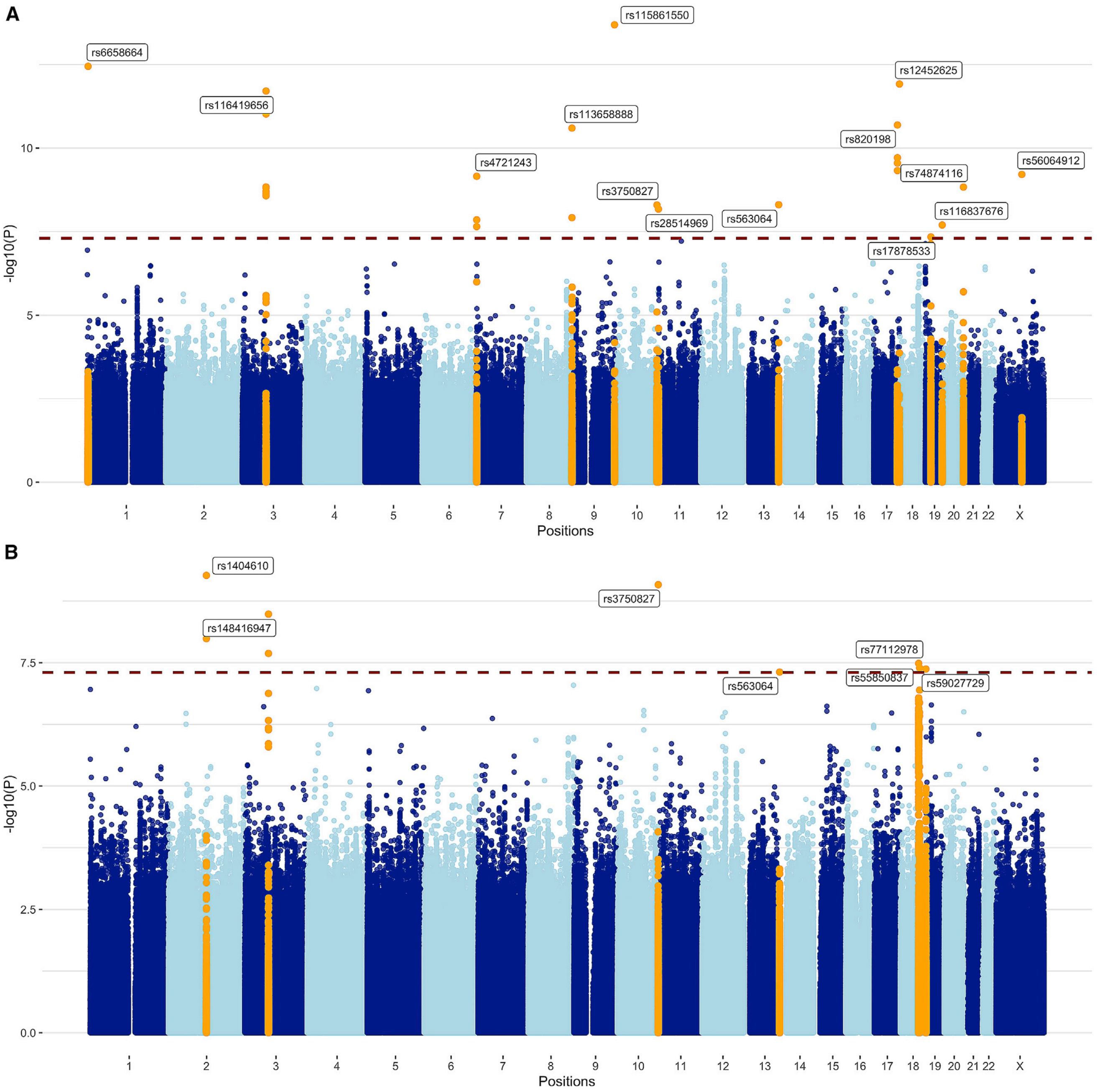

Figure 2. Results from the GWASs of conversion from AS to treatment
(A) in 5,222 prostate cancer (PC) patients of European ancestry; and (B) in discovery and replication cohorts. p values are for variant associations with conversion, adjusted for age and 10 ancestry principal components using Cox proportional hazards models. Blue dashed line denotes the genome-wide significance threshold. Orange peaks indicate genome-wide significant hits ($p < 5 \times 10^{-8}$). The top variants in each chromosome are annotated with their rsID.

associations for *MAST3* (p value = 0.08) and *GAB2* (p value = 4.1×10^{-4}), as well as a suggestive association between imputed expression of the gene *ZNF644* (MIM: 614159) and conversion (p value = 9.9×10^{-5}).

GRS and conversion from AS to treatment

Increasing GRS for PC susceptibility (GRS_{PC}) was positively associated with conversion from AS to treatment, even after adjusting for clinical covariates (Figure 3A; Table S3). The fully adjusted HR for conversion for men in the top decile of the GRS_{PC} compared to the middle two deciles was 1.13 (95% CI, 0.94–1.36; Figure 3A; Table S4). Men in the bottom 10th percentile of the GRS_{PC} distribution had a significantly lower conversion rate than the middle two deciles of the GRS_{PC} (HR = 0.69; 95% CI, 0.56–0.86; Figure 3A; Table S4).

From the 36-variant GRS for PSA concentration (GRS_{PSA}), we observed the opposite pattern: increasing GRS_{PSA} was inversely associated with conversion (Figure 3B; Table S3). Compared to the 40th–60th percentiles, men in the bottom

Table 1. Results for 4 common and 14 rare variants associated with conversion from AS to treatment in a genome-wide association analysis

					HR (95% CI), p value								
rsid	Chr	Genes	Risk allele/ ref allele	RAF	Discovery European			Replication meta-analysis			Combined meta-analysis		
**Common variants**													
rs77112978	18	*ATP8B1* *NEDD4L*[a]	G/C	0.048	1.41	(1.23–1.63)	1.51×10^{-6}	2.24	(1.38–3.65)	1.14×10^{-3}	1.47	(1.28–1.68)	3.26×10^{-8}
rs55850837	18	*MC4R* *CDH20*[a]	A/G	0.050	1.42	(1.24–1.63)	3.43×10^{-7}	1.92	(1.09–3.36)	0.0232	1.45	(1.27–1.65)	4.03×10^{-8}
rs17878533	19	*IL12RB1*[b]	A/G	0.651	1.23	(1.14–1.32)	4.56×10^{-8}	0.94	(0.75–1.18)	0.582	1.2	(1.12–1.28)	4.86×10^{-7}
rs74874116[c]	20	*RBBP8NL* *GATA5*[a]	T/G	0.028	2.67	(1.94–3.66)	1.47×10^{-9}	1.23	(0.93–1.64)	0.146	1.74	(1.41–2.15)	3.14×10^{-7}
**Rare variants**													
rs6658664[c]	1	*PRDM16*[b]	A/G	0.014	5.19	(3.33–8.09)	3.55×10^{-13}	0.94	(0.67–1.33)	0.746	1.79	(1.37–2.46)	2.58×10^{-5}
rs1404610	2	*LINC01101* *GLI2*[a]	G/A	0.012	15.15	(4.64–49.5)	6.69×10^{-6}	3.07	(1.97–4.79)	7.84×10^{-7}	3.74	(2.47–5.67)	5.37×10^{-10}
rs116419656[c]	3	*FAM86DP* *MIR1324*[a]	G/A	0.010	6.89	(4.03–11.8)	1.95×10^{-12}	0.96	(0.47–2.00)	0.923	3.45	(2.24–5.32)	2.06×10^{-8}
rs4721243[c]	7	*MAD1L1*[b]	A/G	0.010	5.65	(3.26–9.8)	7×10^{-10}	0.78	(0.5–1.22)	0.28	1.70	(1.2–2.39)	2.6×10^{-3}
rs113658888[c]	8	*MROH1*[b]	T/C	0.011	3.86	(2.60–5.74)	2.52×10^{-11}	1.22	(0.84–1.78)	0.305	2.11	(1.6–2.77)	9.04×10^{-8}
rs115861550[c]	9	*VAV2*[b]	T/C	0.010	7.51	(4.48–12.6)	2.03×10^{-14}	1.01	(0.69–1.48)	0.943	2.05	(1.51–2.79)	4.26×10^{-6}
rs3750827	10	*EBF3*[b]	A/G	0.011	2.4	(1.79–3.21)	5.05×10^{-9}	1.78	(1.04–3.03)	0.035	2.24	(1.73–2.89)	8.29×10^{-10}
rs28514969[c]	11	*TSPAN4*[b]	C/T	0.011	14.16	(5.78–34.7)	6.69×10^{-9}	1.49	(0.92–2.39)	0.103	2.44	(1.6–3.72)	3.16×10^{-5}
rs563064[c]	13	*F10*[d]	A/G	0.010	34.03	(10.4–111)	4.59×10^{-9}	2.2	(1.45–3.34)	1.96×10^{-4}	2.98	(2.01–4.41)	4.89×10^{-8}
rs820198[c]	17	*RECQL5*[b]	T/C	0.010	14.26	(6.56–31.0)	2.03×10^{-11}	1.2	(0.67–2.17)	0.537	2.97	(1.86–4.75)	5.50×10^{-6}
rs12452625[c]	17	*RFNG*[e]	A/G	0.011	3.84	(2.65–5.57)	1.20×10^{-12}	1.03	(0.68–1.56)	0.878	2.14	(1.62–2.82)	6.84×10^{-8}
rs59027729	19	*HCN2*[b]	A/G	0.014	2.06	(1.58–2.68)	7.24×10^{-8}	1.47	(0.92–2.34)	0.109	1.90	(1.51–2.39)	4.25×10^{-8}
rs116837676[c]	19	*MYADM*[b]	A/G	0.011	5.42	(3.00–9.77)	2.01×10^{-8}	1.72	(1.17–2.53)	5.66×10^{-3}	2.42	(1.76–3.34)	7.34×10^{-8}
rs56064912[c]	X	*POF1B* *MIR1321*[a]	T/G	0.010	24.7	(8.95–68.3)	6.13×10^{-10}	1.07	(0.85–1.36)	0.55	1.26	(1–1.59)	0.0475

Chr, chromosome; Ref, reference; RAF, risk allele frequency; HR, hazard ratio; CI, confidence interval.
Variants included here are those with association $p < 5 \times 10^{-8}$ either in European discovery GWAS or combined meta-analysis of all samples.
[a]Intergenic (genes are two flanking).
[b]Intronic.
[c]Variants with $I^2 > 75\%$.
[d]Upstream.
[e]3′ UTR.

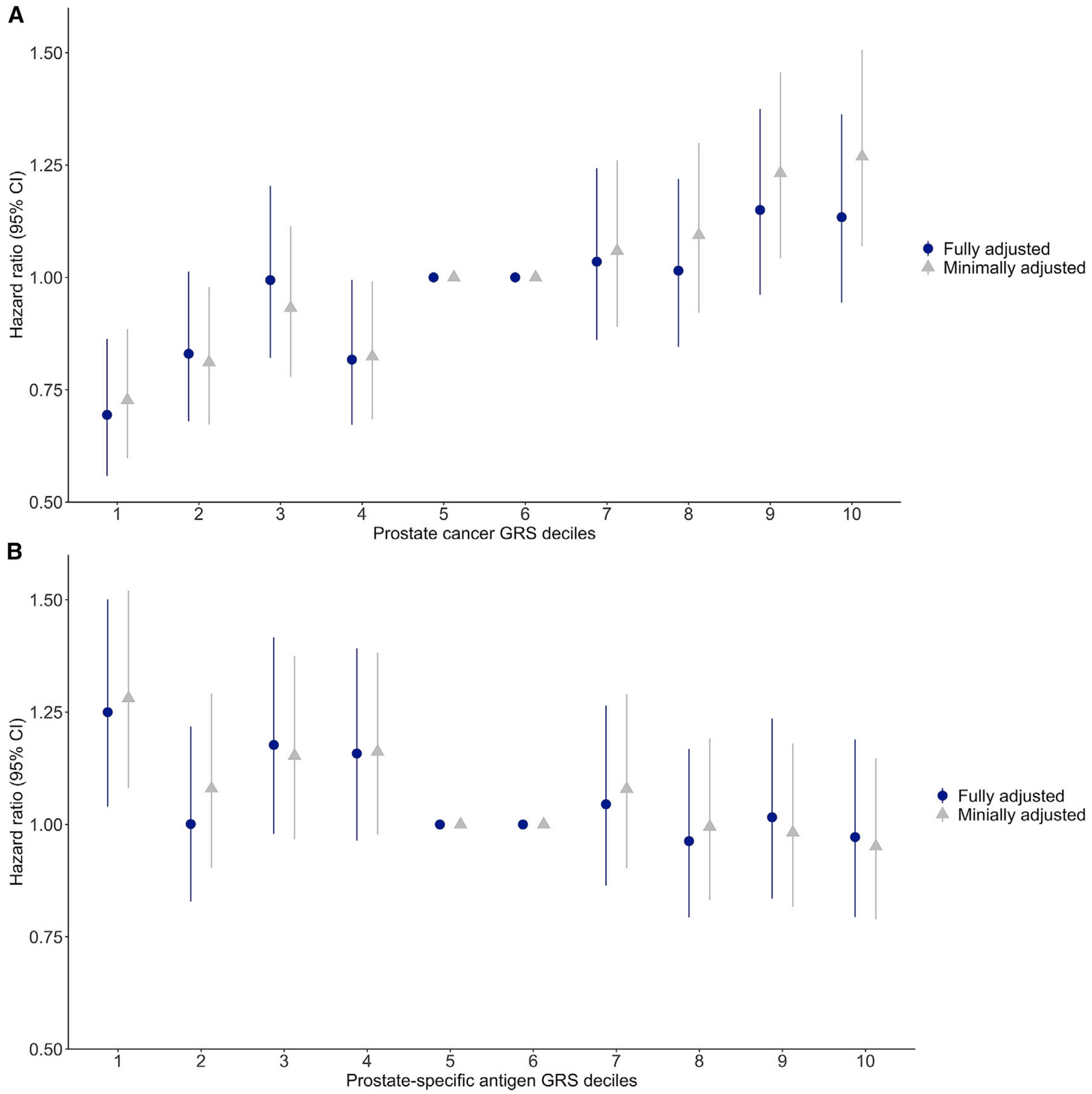

Figure 3. Association between time to conversion from AS to treatment
(A) with the PC genetic risk score (GRS); and (B) with the prostate-specific antigen (PSA) GRS. The fifth and sixth deciles of PC GRSs are used as the reference. Bars indicate 95% confidence intervals (CIs) around the hazard ratio (HR) estimates. The minimally adjusted model includes age and the first 10 genetic principal components. The fully adjusted model also includes Gleason grade group (GG1, GG2, or ≥GG3), PSA concentration (ng/mL), clinical stage (cT1, cT2, or cT3/cT4), and number of positive biopsy cores (1–2, 3, or ≥4).

10th percentile of the PSA GRS distribution experienced a shorter time to conversion (fully adjusted HR = 1.25; 95% CI, 1.04–1.50; Figure 3B; Table S5). For other deciles, both the minimally and fully adjusted models show null associations between the PSA GRS and time to conversion.

Potential clinical utility of the GRS

The time to conversion in the low- and intermediate-risk groups varied depending on whether men were in the top or bottom deciles of the GRS_{PC} and GRS_{PSA} distributions (Figure 4). For GRS_{PC}, the Kaplan-Meier curves contrasting the top versus bottom deciles were significantly different for the low- and intermediate-risk groups ($p = 3 \times 10^{-5}$ and $p = 0.016$, respectively). Similarly, the top and bottom deciles of the GRS_{PSA} differed for the intermediate-risk groups ($p = 0.003$). There was no clear difference between the deciles of GRS_{PC} or GRS_{PSA} in patients with high-risk disease.

A baseline model including age at diagnosis and principal components achieved an AUC = 0.55 for time to conversion. This was substantially improved by incorporating the clinical characteristics into the model: AUC = 0.653 (Table S3). Adding the GRS_{PC} to this model resulted in modest improvement (AUC = 0.659). Augmenting this model with GRS_{PSA} produced minimal improvement (AUC = 0.661).

Discussion

In this GWAS of PC individuals managed with AS, we detected 18 novel variants and two candidate genes

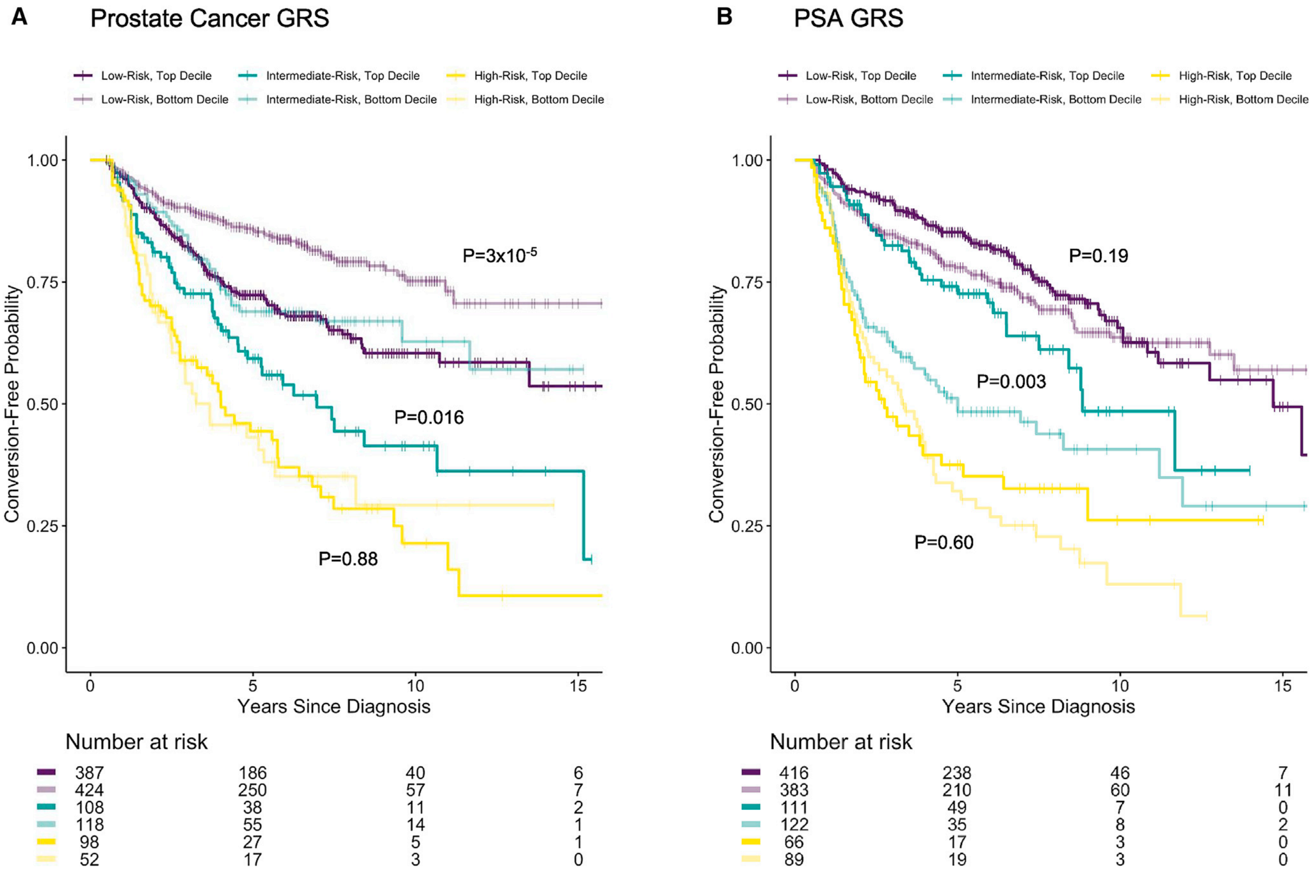

Figure 4. Kaplan-Meier plots of active surveillance conversion-free probability for low, intermediate, and high clinicopathological risk categories

The plots are stratified by the top and bottom deciles of GRSs for PC (GRS_{PC}, A) and for PSA levels (GRS_{PSA}, B). The curves within each risk category are compared between the top and bottom GRS deciles using a log-rank test (p values given next to corresponding curves).

associated with the risk of conversion from AS to treatment. We further found that GRS for PC susceptibility in addition to PSA level were associated with conversion, providing information beyond conventional clinical and pathologic measures of the disease. These findings provide preliminary support for using germline genetic information to inform the initial management of men with newly diagnosed, clinically localized PC.

Of the 18 variants associated with conversion, seven were genome-wide significant in the combined meta-analysis of the discovery and replication samples. This joint analysis affords some gain in statistical power for detecting variant effects while mitigating the "winner's curse" bias inherent to the discovery analysis.[63] Therefore, these seven variants most warrant follow-up association and functional analyses in independent samples. Of the 18 variants associated with conversion, 15 were not previously associated with PC risk. These include a low-frequency (MAF = 0.01) intronic variant, rs4721243 at *MAD1L1*, at a previously identified PC locus.[43] The variant was uncorrelated with the previously reported genome-wide significant PC variant at the locus (rs4513875, $r^2 = 0.012$ in the 1000 Genomes global reference data). One detected variant (rs74874116) was 32 kb away from a PC-associated indel (rs139135938), with little correlation ($r^2 = 0.015$ in 1000 Genomes). The neighboring gene, *GATA5*, encodes a transcription factor that contains two GATA-type zinc fingers and is required during cardiovascular development.[64] This gene contains two variants previously associated with benign prostatic hyperplasia (MIM: 600082) and associated lower urinary tract symptoms[65] (MIM: 618612). Another variant in a PC risk locus was rs1404610, nearby *GLI2*, a transcription factor that one study found regulates the growth and tumorigenicity of prostate cells.[66]

Many of the novel variants we found to be associated with conversion are intronic, including variants in genes involved in cellular signaling, growth, and differentiation. *PRDM16* (MIM: 605557), where rs6658664 is located, is associated with evasion of apoptosis by prostatic cancer cells.[67] Intronic variant rs115861550 in *VAV2* (MIM: 600428) is upregulated in human PC tumors and is a prognostic indicator for poor outcome.[68] Another intronic variant, *EBF3* (MIM: 607407), has been shown to regulate the expression of genes involved in cell growth, proliferation, and apoptosis.[69] *RECQL5* (MIM: 603781), where variant rs820198 is located, regulates DNA repair intermediate structures, and studies have observed elevated *RECQL5* expression in other cancers such as breast (MIM:

114480) and bladder (MIM: 109800).[70–72] Variant rs820198 is annotated to an active CTCF (CCCTC-binding factor, MIM: 604167) binding site, and CTCF expression is linked to poor outcomes in PC.[73] Although intergenic, variant rs77112978 is near *NEDD4L* (MIM: 606384), whose expression is decreased in PC.[74] Intergenic variant rs55850837-A, associated with conversion in our study, was associated with reduced body mass index[75] and body fat percentage[76] in the phenome-wide association data curated by the IEU OpenGWAS Project.[77] Variant rs12452625, a 3′ UTR variant of *RFNG* (MIM: 602578) gene, is correlated with variants associated with multiple traits, including heel bone mineral density, lung function, and waist-hip ratio.[78,79] This variant is also predicted to be a functional target of microRNA hsa-miR-629-3p, which may serve as a biomarker for lung metastases of triple-negative breast cancer.[80]

Our TWAS suggests a possible role for *MAST3* and *GAB2* in conversion. A study described *MAST3* as an inflammatory bowel disease (IBD, MIM: 601458) susceptibility gene that regulates NF-κB (MIM: 164011) activity through TLR4[81] (MIM: 603030). Two recent studies have described increased risk for PC in men with IBD.[82,83] Regarding *GAB2*, the knockdown of this gene in PC cells altered the expression of over 1,200 genes and inhibited p53 signaling.[84]

From either the minimally or the fully adjusted models where the PS GRS was continuous, we found that the PC GRS based on 269 known risk variants was positively associated with conversion. Moreover, a continuous PSA GRS based on 36 known genetic variants for PSA levels exhibited a modest but statistically significant inverse association with conversion. We expected these GRSs to have opposite directions of effect on conversion, given that the PSA GRS may reflect the potential ascertainment of higher-risk PC in men with lower genetically predicted PSA levels. While the overall GRS only contributed modest model discrimination beyond established risk factors for conversion (i.e., Gleason grade group, stage, and the number of positive biopsy cores), the associations observed in the tails (i.e., deciles) of the GRS distribution were most pronounced among men in low- and intermediate-risk clinicopathological categories. This finding suggests an increased utility of genetic information for men with lower-risk disease, but high PC GRS (or low PSA GRS), who may be more likely candidates for early treatment or possibly a higher-intensity of surveillance. Moreover, this also suggests potential clinical utility from incorporating genetic information into prediction models composed of many of the same AS outcome risk factors considered here (e.g., the Canary model).[85] A recent study of European ancestry men with low-risk PC managed on AS reported associations between higher PC GRS with more positive cores and with bilateral tumor location at diagnostic and surveillance biopsy;[86] note that ~50% of the men in this previous study are also included here, comprising ~10% of our study population. Despite the individual deciles of the PSA GRS showing a decreasing trend, only the lowest 0%–10% GRS category showed a statistically significant association with AS conversion. This suggests that the PSA GRS used here based on a small number of genetic variants may have limited clinical utility to evaluate the risk of conversion. This may be improved with future PSA GRSs comprised of larger numbers of variants.

Strengths of this study include leveraging a large, multi-institutional collaborative study of AS to model the effects of genetic risk variants independent of clinical risk parameters. Sixty-three percent of the replication sample ($n = 714$) were men of non-European genetic ancestry, allowing us to test the generalizability of the variants discovered in the European sample. Our GRS included the most recently available GWAS weights from PC and PSA. Limitations of our study included the lack of confirmatory or surveillance biopsies to reduce misclassification of clinical parameters at diagnosis and follow-up. In addition, conversion could conflate disease progression with patient anxiety and/or physician preference for AS management. However, discontinuing AS due to anxiety was relatively uncommon in this study (about 6% of events), and these individuals were censored in the GWAS analysis. Furthermore, given the relatively short follow-up for more robust PC outcomes, the sample sizes for PSA failure after treatment ($n = 124$), metastases ($n = 29$), or PC-specific death ($n = 11$) are too small for a GWAS analysis. Most of the variants with AS conversion had low MAF, which contrasts with findings of more common variants associated with risk of PC. While this may reflect something unique about the genetic etiology of AS conversion, it may also reflect our ability to detect associations given our cohort sample size. Due to the limited sample size in some of the contributing studies, we did not adjust for or meta-analyze by individual study in the association analyses. Nevertheless, we do not think confounding by study site is a concern, because the data genotyped by CIDR were run and analyzed together on a single array, the discovery sample was restricted to individuals of European ancestry with adjustment for principal components, and the relationship between clinical factors and time to AS conversion observed in our study agrees with previous publications.[49] In addition, we observed some large I^2 for the rare variants, indicating heterogeneity between the discovery and replication analyses. The inconsistency and between-study heterogeneity may be due to real differences or biases in the genetic effects across populations. Further research will be required to confirm the associations reported here.

In summary, we have undertaken the first GWAS of conversion among men diagnosed with PC. This multi-institutional study detected a genetic basis of conversion, suggesting that genetic factors may provide valuable information to stratify men with PC by their risk of discontinuing AS. Important future work will expand this study to more men placed on AS, increasing our ability to detect genetic variants associated with conversion. This may in turn help address concerns that biopsy sampling may underestimate a tumor's aggressiveness and provide a more personalized approach to decisions surrounding AS.

Data availability

The CIDR genotyped data analyzed in this publication have been deposited in dbGap and are accessible through dbGap study accession number phs002056.v1.p1. The MD Anderson data are available via request to the PRACTICAL consortium.

Supplemental information

Supplemental information can be found online at https://doi.org/10.1016/j.xhgg.2021.100070.

Acknowledgments

This work was supported by National Institutes of Health (NIH) grant P50CA180995 (W.J.C.) 08/01/15–07/31/20 NIH/NCI SPORE in Prostate Cancer PI on the SPORE grant, Center for Inherited Disease Research (CIDR) award from the NCI for patient sample genotyping (X01HG009642), CIDR contract no. HHSN2682017 00006I, the Urological Research Foundation (W.J.C.), and the NorthShore University Health System, Evanston, IL, USA (B.T.H.). Additional support was provided by award numbers R01CA158627 and R01CA195505 (L.S.M.), P50 CA186786 (T.M.M.), UL1 TR000445 from NCATS/NIH for Vanderbilt REDCap (D.A.B.), P50 CA097186 and K05 CA175147 (J.L.S.), and U01 CA113913 (M.G.S.) from the NIH, and W81XWH-13-2-0074 (P.R.C.) from the Department of Defense.

Declaration of interests

The authors declare no competing interests.

Received: August 20, 2021
Accepted: November 12, 2021

Web resources

IEU OpenGWAS Project, https://gwas.mrcieu.ac.uk/
METAL, https://genome.sph.umich.edu/wiki/METAL_Documentation
MetaXcan, S-MetaXcan, https://github.com/hakyimlab/MetaXcan
OMIM, https://www.omim.org/
PLINK v.1.9, https://www.cog-genomics.org/plink/
PRACTICAL consortium, http://practical.icr.ac.uk/blog/

References

1. Washington, S.L., 3rd, Jeong, C.W., Lonergan, P.E., Herlemann, A., Gomez, S.L., Carroll, P.R., and Cooperberg, M.R. (2020). Regional Variation in Active Surveillance for Low-Risk Prostate Cancer in the US. JAMA Netw. Open *3*, e2031349.
2. Mohler, J.L., Antonarakis, E.S., Armstrong, A.J., D'Amico, A.V., Davis, B.J., Dorff, T., Eastham, J.A., Enke, C.A., Farrington, T.A., Higano, C.S., et al. (2019). Prostate Cancer, Version 2.2019, NCCN Clinical Practice Guidelines in Oncology. J. Natl. Compr. Canc. Netw. *17*, 479–505.
3. Loeb, S., Byrne, N., Makarov, D.V., Lepor, H., and Walter, D. (2018). Use of Conservative Management for Low-Risk Prostate Cancer in the Veterans Affairs Integrated Health Care System From 2005-2015. JAMA *319*, 2231–2233.
4. Parikh, R.B., Robinson, K.W., Chhatre, S., Medvedeva, E., Cashy, J.P., Veera, S., Bauml, J.M., Fojo, T., Navathe, A.S., Malkowicz, S.B., et al. (2020). Comparison by Race of Conservative Management for Low-Risk and Intermediate-Risk Prostate Cancers in Veterans From 2004 to 2018. JAMA Netw. Open *3*, e2018318.
5. Loeb, S., Folkvaljon, Y., Curnyn, C., Robinson, D., Bratt, O., and Stattin, P. (2017). Uptake of Active Surveillance for Very-Low-Risk Prostate Cancer in Sweden. JAMA Oncol. *3*, 1393–1398.
6. Stolzenbach, L.F., Rosiello, G., Pecoraro, A., Palumbo, C., Luzzago, S., Deuker, M., Tian, Z., Knipper, A.-S., Pompe, R., Zorn, K.C., et al. (2020). Prostate Cancer Grade and Stage Misclassification in Active Surveillance Candidates: Black Versus White Patients. J. Natl. Compr. Canc. Netw. *18*, 1492–1499.
7. Klotz, L., Vesprini, D., Sethukavalan, P., Jethava, V., Zhang, L., Jain, S., Yamamoto, T., Mamedov, A., and Loblaw, A. (2015). Long-term follow-up of a large active surveillance cohort of patients with prostate cancer. J. Clin. Oncol. *33*, 272–277.
8. Cooperberg, M.R., Brooks, J.D., Faino, A.V., Newcomb, L.F., Kearns, J.T., Carroll, P.R., Dash, A., Etzioni, R., Fabrizio, M.D., Gleave, M.E., et al. (2018). Refined Analysis of Prostate-specific Antigen Kinetics to Predict Prostate Cancer Active Surveillance Outcomes. Eur. Urol. *74*, 211–217.
9. Williams, C., Khondakar, N.R., Daneshvar, M.A., O'Connor, L.P., Gomella, P.T., Mehralivand, S., Yerram, N.K., Egan, J., Gurram, S., Rompré-Brodeur, A., et al. (2021). The Risk of Prostate Cancer Progression in Active Surveillance Patients with Bilateral Disease Detected by Combined Magnetic Resonance Imaging-Fusion and Systematic Biopsy. J. Urol *206*, 1157–1165.
10. Eggener, S.E., Rumble, R.B., Armstrong, A.J., Morgan, T.M., Crispino, T., Cornford, P., van der Kwast, T., Grignon, D.J., Rai, A.J., Agarwal, N., et al. (2020). Molecular Biomarkers in Localized Prostate Cancer: ASCO Guideline. J. Clin. Oncol. *38*, 1474–1494.
11. Black, M.H., Li, S., LaDuca, H., Lo, M.T., Chen, J., Hoiness, R., Gutierrez, S., Tippin-Davis, B., Lu, H.M., Gielzak, M., et al. (2020). Validation of a prostate cancer polygenic risk score. Prostate *80*, 1314–1321.
12. Langeberg, W.J., Isaacs, W.B., and Stanford, J.L. (2007). Genetic etiology of hereditary prostate cancer. Front. Biosci. *12*, 4101–4110.
13. Lichtenstein, P., Holm, N.V., Verkasalo, P.K., Iliadou, A., Kaprio, J., Koskenvuo, M., Pukkala, E., Skytthe, A., and Hemminki, K. (2000). Environmental and heritable factors in the causation of cancer–analyses of cohorts of twins from Sweden, Denmark, and Finland. N. Engl. J. Med. *343*, 78–85.
14. Narod, S. (1999). Genetic epidemiology of prostate cancer. Biochim. Biophys. Acta *1423*, F1–F13.
15. Schumacher, F.R., Al Olama, A.A., Berndt, S.I., Benlloch, S., Ahmed, M., Saunders, E.J., Dadaev, T., Leongamornlert, D., Anokian, E., Cieza-Borrella, C., et al.; Profile Study; Australian Prostate Cancer BioResource (APCB); IMPACT Study; Canary PASS Investigators; Breast and Prostate Cancer Cohort Consortium (BPC3); PRACTICAL (Prostate Cancer Association Group to Investigate Cancer-Associated Alterations in the Genome) Consortium; Cancer of the Prostate in Sweden (CAPS); Prostate Cancer Genome-wide Association Study of Uncommon Susceptibility Loci (PEGASUS); and Genetic Associations and Mechanisms in Oncology (GAME-ON)/Elucidating Loci Involved in Prostate Cancer Susceptibility

(ELLIPSE) Consortium (2018). Association analyses of more than 140,000 men identify 63 new prostate cancer susceptibility loci. Nat. Genet. *50*, 928–936.

16. Ahn, J., Kibel, A.S., Park, J.Y., Rebbeck, T.R., Rennert, H., Stanford, J.L., Ostrander, E.A., Chanock, S., Wang, M.-H., Mittal, R.D., et al. (2011). Prostate cancer predisposition loci and risk of metastatic disease and prostate cancer recurrence. Clin. Cancer Res. *17*, 1075–1081.
17. Amin Al Olama, A., Kote-Jarai, Z., Schumacher, F.R., Wiklund, F., Berndt, S.I., Benlloch, S., Giles, G.G., Severi, G., Neal, D.E., Hamdy, F.C., et al.; UK Genetic Prostate Cancer Study Collaborators/British Association of Urological Surgeons' Section of Oncology; UK ProtecT Study Collaborators; Australian Prostate Cancer Bioresource; and PRACTICAL Consortium (2013). A meta-analysis of genome-wide association studies to identify prostate cancer susceptibility loci associated with aggressive and non-aggressive disease. Hum. Mol. Genet. *22*, 408–415.
18. Bao, B.-Y., Pao, J.-B., Huang, C.-N., Pu, Y.-S., Chang, T.-Y., Lan, Y.-H., Lu, T.-L., Lee, H.-Z., Chen, L.-M., Ting, W.-C., et al. (2012). Significant associations of prostate cancer susceptibility variants with survival in patients treated with androgen-deprivation therapy. Int. J. Cancer *130*, 876–884.
19. Bensen, J.T., Xu, Z., Smith, G.J., Mohler, J.L., Fontham, E.T.H., and Taylor, J.A. (2013). Genetic polymorphism and prostate cancer aggressiveness: a case-only study of 1,536 GWAS and candidate SNPs in African-Americans and European-Americans. Prostate *73*, 11–22.
20. Chen, M., Huang, Y.-C., Yang, S., Hsu, J.-M., Chang, Y.-H., Huang, W.J.-S., and Chen, Y.-M.A. (2010). Common variants at 8q24 are associated with prostate cancer risk in Taiwanese men. Prostate *70*, 502–507.
21. Eeles, R.A., Kote-Jarai, Z., Giles, G.G., Olama, A.A.A., Guy, M., Jugurnauth, S.K., Mulholland, S., Leongamornlert, D.A., Edwards, S.M., Morrison, J., et al.; UK Genetic Prostate Cancer Study Collaborators; British Association of Urological Surgeons' Section of Oncology; and UK ProtecT Study Collaborators (2008). Multiple newly identified loci associated with prostate cancer susceptibility. Nat. Genet. *40*, 316–321.
22. Eeles, R.A., Olama, A.A.A., Benlloch, S., Saunders, E.J., Leongamornlert, D.A., Tymrakiewicz, M., Ghoussaini, M., Luccarini, C., Dennis, J., Jugurnauth-Little, S., et al. (2013). Identification of 23 new prostate cancer susceptibility loci using the iCOGS custom genotyping array. Nat Genet *45*, 385–391, 391e1–2.
23. FitzGerald, L.M., Kwon, E.M., Conomos, M.P., Kolb, S., Holt, S.K., Levine, D., Feng, Z., Ostrander, E.A., and Stanford, J.L. (2011). Genome-wide association study identifies a genetic variant associated with risk for more aggressive prostate cancer. Cancer Epidemiol. Biomarkers Prev. *20*, 1196–1203.
24. Gudmundsson, J., Sulem, P., Rafnar, T., Bergthorsson, J.T., Manolescu, A., Gudbjartsson, D., Agnarsson, B.A., Sigurdsson, A., Benediktsdottir, K.R., Blondal, T., et al. (2008). Common sequence variants on 2p15 and Xp11.22 confer susceptibility to prostate cancer. Nat. Genet. *40*, 281–283.
25. Gudmundsson, J., Besenbacher, S., Sulem, P., Gudbjartsson, D.F., Olafsson, I., Arinbjarnarson, S., Agnarsson, B.A., Benediktsdottir, K.R., Isaksson, H.J., Kostic, J.P., et al. (2010). Genetic correction of PSA values using sequence variants associated with PSA levels. Sci. Transl. Med. *2*, 62ra92.
26. Helfand, B.T., Loeb, S., Cashy, J., Meeks, J.J., Thaxton, C.S., Han, M., and Catalona, W.J. (2008). Tumor characteristics of carriers and noncarriers of the deCODE 8q24 prostate cancer susceptibility alleles. J. Urol. *179*, 2197–2201, discussion 2202.
27. Helfand, B.T., Kan, D., Modi, P., and Catalona, W.J. (2011). Prostate cancer risk alleles significantly improve disease detection and are associated with aggressive features in patients with a "normal" prostate specific antigen and digital rectal examination. Prostate *71*, 394–402.
28. Lange, E.M., Johnson, A.M., Wang, Y., Zuhlke, K.A., Lu, Y., Ribado, J.V., Keele, G.R., Li, J., Duan, Q., Li, G., et al. (2014). Genome-wide association scan for variants associated with early-onset prostate cancer. PLoS ONE *9*, e93436.
29. Lange, E.M., Salinas, C.A., Zuhlke, K.A., Ray, A.M., Wang, Y., Lu, Y., Ho, L.A., Luo, J., and Cooney, K.A. (2012). Early onset prostate cancer has a significant genetic component. Prostate *72*, 147–156.
30. Lin, D.W., FitzGerald, L.M., Fu, R., Kwon, E.M., Zheng, S.L., Kolb, S., Wiklund, F., Stattin, P., Isaacs, W.B., Xu, J., et al. (2011). Genetic variants in the LEPR, CRY1, RNASEL, IL4, and ARVCF genes are prognostic markers of prostate cancer-specific mortality. Cancer Epidemiol. Biomarkers Prev. *20*, 1928–1936.
31. Loeb, S., Carter, H.B., Walsh, P.C., Isaacs, W.B., Kettermann, A., Tanaka, T., Ferrucci, L., and Metter, E.J. (2009). Single nucleotide polymorphisms and the likelihood of prostate cancer at a given prostate specific antigen level. J. Urol. *182*, 101–104, discussion 105.
32. Pal, P., Xi, H., Guha, S., Sun, G., Helfand, B.T., Meeks, J.J., Suarez, B.K., Catalona, W.J., and Deka, R. (2009). Common variants in 8q24 are associated with risk for prostate cancer and tumor aggressiveness in men of European ancestry. Prostate *69*, 1548–1556.
33. Pomerantz, M.M., Werner, L., Xie, W., Regan, M.M., Lee, G.-S.M., Sun, T., Evan, C., Petrozziello, G., Nakabayashi, M., Oh, W.K., et al. (2011). Association of prostate cancer risk Loci with disease aggressiveness and prostate cancer-specific mortality. Cancer Prev. Res. (Phila.) *4*, 719–728.
34. Sun, J., Lange, E.M., Isaacs, S.D., Liu, W., Wiley, K.E., Lange, L., Gronberg, H., Duggan, D., Carpten, J.D., Walsh, P.C., et al. (2008). Chromosome 8q24 risk variants in hereditary and non-hereditary prostate cancer patients. Prostate *68*, 489–497.
35. Xu, J., Zheng, S.L., Isaacs, S.D., Wiley, K.E., Wiklund, F., Sun, J., Kader, A.K., Li, G., Purcell, L.D., Kim, S.-T., et al. (2010). Inherited genetic variant predisposes to aggressive but not indolent prostate cancer. Proc. Natl. Acad. Sci. USA *107*, 2136–2140.
36. Goh, C.L., and Eeles, R.A. (2014). Germline genetic variants associated with prostate cancer and potential relevance to clinical practice. Recent Results Cancer Res. *202*, 9–26.
37. Ishak, M.B., and Giri, V.N. (2011). A systematic review of replication studies of prostate cancer susceptibility genetic variants in high-risk men originally identified from genome-wide association studies. Cancer Epidemiol. Biomarkers Prev. *20*, 1599–1610.
38. Kim, S.-T., Cheng, Y., Hsu, F.-C., Jin, T., Kader, A.K., Zheng, S.L., Isaacs, W.B., Xu, J., and Sun, J. (2010). Prostate cancer risk-associated variants reported from genome-wide association studies: meta-analysis and their contribution to genetic Variation. Prostate *70*, 1729–1738.
39. Lindström, S., Zheng, S.L., Wiklund, F., Jonsson, B.-A., Adami, H.-O., Bälter, K.A., Brookes, A.J., Sun, J., Chang, B.-L., Liu, W., et al. (2006). Systematic replication study of reported genetic associations in prostate cancer: Strong support for genetic variation in the androgen pathway. Prostate *66*, 1729–1743.

40. Pomerantz, M.M., and Freedman, M.L. (2010). Genetics of prostate cancer risk. Mt. Sinai J. Med. *77*, 643–654.
41. Na, R., Liu, F., Zhang, P., Ye, D., Xu, C., Shao, Q., Qi, J., Wang, X., Chen, Z., Wang, M., et al. (2013). Evaluation of reported prostate cancer risk-associated SNPs from genome-wide association studies of various racial populations in Chinese men. Prostate *73*, 1623–1635.
42. Bensen, J.T., Xu, Z., McKeigue, P.M., Smith, G.J., Fontham, E.T.H., Mohler, J.L., and Taylor, J.A. (2014). Admixture mapping of prostate cancer in African Americans participating in the North Carolina-Louisiana Prostate Cancer Project (PCaP). Prostate *74*, 1–9.
43. Conti, D.V., Darst, B.F., Moss, L.C., Saunders, E.J., Sheng, X., Chou, A., Schumacher, F.R., Olama, A.A.A., Benlloch, S., Dadaev, T., et al. (2021). Trans-ancestry genome-wide association meta-analysis of prostate cancer identifies new susceptibility loci and informs genetic risk prediction. Nat. Genet. *53*, 65–75.
44. Chen, H., Liu, X., Brendler, C.B., Ankerst, D.P., Leach, R.J., Goodman, P.J., Lucia, M.S., Tangen, C.M., Wang, L., Hsu, F.-C., et al. (2016). Adding genetic risk score to family history identifies twice as many high-risk men for prostate cancer: Results from the prostate cancer prevention trial. Prostate *76*, 1120–1129.
45. Helfand, B.T., Kearns, J., Conran, C., and Xu, J. (2016). Clinical validity and utility of genetic risk scores in prostate cancer. Asian J. Androl. *18*, 509–514.
46. Huynh-Le, M.-P., Karunamuni, R., Fan, C.C., Thompson, W.K., Muir, K., Lophatananon, A., Tye, K., Wolk, A., Håkansson, N., Mills, I.G., et al.; PRACTICAL Consortium (2021). Common genetic and clinical risk factors: association with fatal prostate cancer in the Cohort of Swedish Men. Prostate Cancer Prostatic Dis. *24*, 845–851.
47. Helfand, B.T., and Xu, J. (2021). Germline Testing for Prostate Cancer Prognosis: Implications for Active Surveillance. Urol. Clin. North Am. *48*, 401–409.
48. Hoffmann, T.J., Passarelli, M.N., Graff, R.E., Emami, N.C., Sakoda, L.C., Jorgenson, E., Habel, L.A., Shan, J., Ranatunga, D.K., Quesenberry, C.P., et al. (2017). Genome-wide association study of prostate-specific antigen levels identifies novel loci independent of prostate cancer. Nat. Commun. *8*, 14248.
49. Cooley, L.F., Emeka, A.A., Meyers, T.J., Helfand, B.T., Scholtens, D.M., Witte, J.S., and Catalona, W.J. (2021). Factors Associated with Time to Conversion from Active Surveillance to Treatment for Prostate Cancer in a Multi-institutional Cohort. J. Urol *206*, 1147–1156.
50. Van Hemelrijck, M., Ji, X., Helleman, J., Roobol, M.J., van der Linden, W., Nieboer, D., Bangma, C.H., Frydenberg, M., Rannikko, A., Lee, L.S., et al.; Members of the Movember Foundation's Global Action Plan Prostate Cancer Active Surveillance GAP3 consortium; and Members of the Movember Foundation's Global Action Plan Prostate Cancer Active Surveillance GAP3 consortium (2019). Reasons for Discontinuing Active Surveillance: Assessment of 21 Centres in 12 Countries in the Movember GAP3 Consortium. Eur. Urol. *75*, 523–531.
51. Gregg, J.R., Davis, J.W., Reichard, C., Wang, X., Achim, M., Chapin, B.F., Pisters, L., Pettaway, C., Ward, J.F., Choi, S., et al. (2020). Determining Clinically Based Factors Associated With Reclassification in the Pre-MRI Era using a Large Prospective Active Surveillance Cohort. Urology *138*, 91–97.
52. Welty, C.J., Cowan, J.E., Nguyen, H., Shinohara, K., Perez, N., Greene, K.L., Chan, J.M., Meng, M.V., Simko, J.P., Cooperberg, M.R., and Carroll, P.R. (2015). Extended followup and risk factors for disease reclassification in a large active surveillance cohort for localized prostate cancer. J. Urol. *193*, 807–811.
53. Hamdy, F.C., Donovan, J.L., Lane, J.A., Mason, M., Metcalfe, C., Holding, P., Davis, M., Peters, T.J., Turner, E.L., Martin, R.M., et al.; ProtecT Study Group (2016). 10-Year Outcomes after Monitoring, Surgery, or Radiotherapy for Localized Prostate Cancer. N. Engl. J. Med. *375*, 1415–1424.
54. Harris, P.A., Taylor, R., Thielke, R., Payne, J., Gonzalez, N., and Conde, J.G. (2009). Research electronic data capture (REDCap)–a metadata-driven methodology and workflow process for providing translational research informatics support. J. Biomed. Inform. *42*, 377–381.
55. Harris, P.A., Taylor, R., Minor, B.L., Elliott, V., Fernandez, M., O'Neal, L., McLeod, L., Delacqua, G., Delacqua, F., Kirby, J., Duda, S.N.; and REDCap Consortium (2019). The REDCap consortium: Building an international community of software platform partners. J. Biomed. Inform. *95*, 103208.
56. Gordetsky, J., and Epstein, J. (2016). Grading of prostatic adenocarcinoma: current state and prognostic implications. Diagn. Pathol. *11*, 25.
57. Alexander, D.H., Novembre, J., and Lange, K. (2009). Fast model-based estimation of ancestry in unrelated individuals. Genome Res. *19*, 1655–1664.
58. Emami, N.C., Leong, L., Wan, E., Van Blarigan, E.L., Cooperberg, M.R., Tenggara, I., Carroll, P.R., Chan, J.M., Witte, J.S., and Simko, J.P. (2017). Tissue Sources for Accurate Measurement of Germline DNA Genotypes in Prostate Cancer Patients Treated With Radical Prostatectomy. Prostate *77*, 425–434.
59. Rizvi, A.A., Karaesmen, E., Morgan, M., Preus, L., Wang, J., Sovic, M., Hahn, T., and Sucheston-Campbell, L.E. (2019). gwasurvivr: an R package for genome-wide survival analysis. Bioinformatics *35*, 1968–1970.
60. Willer, C.J., Li, Y., and Abecasis, G.R. (2010). METAL: fast and efficient meta-analysis of genomewide association scans. Bioinformatics *26*, 2190–2191.
61. Barbeira, A.N., Pividori, M., Zheng, J., Wheeler, H.E., Nicolae, D.L., and Im, H.K. (2019). Integrating predicted transcriptome from multiple tissues improves association detection. PLoS Genet. *15*, e1007889.
62. Chambless, L.E., and Diao, G. (2006). Estimation of time-dependent area under the ROC curve for long-term risk prediction. Stat. Med. *25*, 3474–3486.
63. Palmer, C., and Pe'er, I. (2017). Statistical correction of the Winner's Curse explains replication variability in quantitative trait genome-wide association studies. PLoS Genet. *13*, e1006916.
64. Reiter, J.F., Alexander, J., Rodaway, A., Yelon, D., Patient, R., Holder, N., and Stainier, D.Y.R. (1999). Gata5 is required for the development of the heart and endoderm in zebrafish. Genes Dev. *13*, 2983–2995.
65. Gudmundsson, J., Sigurdsson, J.K., Stefansdottir, L., Agnarsson, B.A., Isaksson, H.J., Stefansson, O.A., Gudjonsson, S.A., Gudbjartsson, D.F., Masson, G., Frigge, M.L., et al. (2018). Genome-wide associations for benign prostatic hyperplasia reveal a genetic correlation with serum levels of PSA. Nat. Commun. *9*, 4568.
66. Thiyagarajan, S., Bhatia, N., Reagan-Shaw, S., Cozma, D., Thomas-Tikhonenko, A., Ahmad, N., and Spiegelman, V.S. (2007). Role of GLI2 transcription factor in growth and tumorigenicity of prostate cells. Cancer Res. *67*, 10642–10646.

67. Zhu, S., Xu, Y., Song, M., Chen, G., Wang, H., Zhao, Y., Wang, Z., and Li, F. (2016). PRDM16 is associated with evasion of apoptosis by prostatic cancer cells according to RNA interference screening. Mol. Med. Rep. *14*, 3357–3361.
68. Magani, F., Peacock, S.O., Rice, M.A., Martinez, M.J., Greene, A.M., Magani, P.S., Lyles, R., Weitz, J.R., and Burnstein, K.L. (2017). Targeting AR Variant-Coactivator Interactions to Exploit Prostate Cancer Vulnerabilities. Mol. Cancer Res. *15*, 1469–1480.
69. Zhao, L.Y., Niu, Y., Santiago, A., Liu, J., Albert, S.H., Robertson, K.D., and Liao, D. (2006). An EBF3-mediated transcriptional program that induces cell cycle arrest and apoptosis. Cancer Res. *66*, 9445–9452.
70. Arora, A., Abdel-Fatah, T.M.A., Agarwal, D., Doherty, R., Croteau, D.L., Moseley, P.M., Hameed, K., Green, A., Aleskandarany, M.A., Rakha, E.A., et al. (2016). Clinicopathological and prognostic significance of RECQL5 helicase expression in breast cancers. Carcinogenesis *37*, 63–71.
71. Chen, E., Ahn, J.S., Sykes, D.B., Breyfogle, L.J., Godfrey, A.L., Nangalia, J., Ko, A., DeAngelo, D.J., Green, A.R., and Mullally, A. (2015). RECQL5 Suppresses Oncogenic JAK2-Induced Replication Stress and Genomic Instability. Cell Rep. *13*, 2345–2352.
72. Patterson, K., Arya, L., Bottomley, S., Morgan, S., Cox, A., Catto, J., and Bryant, H.E. (2016). Altered RECQL5 expression in urothelial bladder carcinoma increases cellular proliferation and makes RECQL5 helicase activity a novel target for chemotherapy. Oncotarget *7*, 76140–76150.
73. Höflmayer, D., Steinhoff, A., Hube-Magg, C., Kluth, M., Simon, R., Burandt, E., Tsourlakis, M.C., Minner, S., Sauter, G., Büscheck, F., et al. (2020). Expression of CCCTC-binding factor (CTCF) is linked to poor prognosis in prostate cancer. Mol. Oncol. *14*, 129–138.
74. Hu, X.Y., Xu, Y.M., Fu, Q., Yu, J.J., and Huang, J. (2009). Nedd4L expression is downregulated in prostate cancer compared to benign prostatic hyperplasia. Eur. J. Surg. Oncol. *35*, 527–531.
75. Biobank, U.K.. https://www.ukbiobank.ac.uk/.
76. Elsworth, B.L. (2017). MRC IEU UK Biobank GWAS pipeline version 1. https://research-information.bris.ac.uk/en/datasets/mrc-ieu-uk-biobank-gwas-pipeline-version-1.
77. Elsworth, B., Lyon, M., Alexander, T., Liu, Y., Matthews, P., Hallett, J., Bates, P., Palmer, T., Haberland, V., Smith, G.D., et al. (2020). The MRC IEU OpenGWAS data infrastructure (Genetics). https://gwas.mrcieu.ac.uk/about/.
78. Kichaev, G., Bhatia, G., Loh, P.-R., Gazal, S., Burch, K., Freund, M.K., Schoech, A., Pasaniuc, B., and Price, A.L. (2019). Leveraging Polygenic Functional Enrichment to Improve GWAS Power. Am. J. Hum. Genet. *104*, 65–75.
79. Lotta, L.A., Wittemans, L.B.L., Zuber, V., Stewart, I.D., Sharp, S.J., Luan, J., Day, F.R., Li, C., Bowker, N., Cai, L., et al. (2018). Association of Genetic Variants Related to Gluteofemoral vs Abdominal Fat Distribution With Type 2 Diabetes, Coronary Disease, and Cardiovascular Risk Factors. JAMA *320*, 2553–2563.
80. Wang, J., Song, C., Tang, H., Zhang, C., Tang, J., Li, X., Chen, B., and Xie, X. (2017). miR-629-3p may serve as a novel biomarker and potential therapeutic target for lung metastases of triple-negative breast cancer. Breast Cancer Res. *19*, 72.
81. Labbé, C., Goyette, P., Lefebvre, C., Stevens, C., Green, T., Tello-Ruiz, M.K., Cao, Z., Landry, A.L., Stempak, J., Annese, V., et al. (2008). MAST3: a novel IBD risk factor that modulates TLR4 signaling. Genes Immun. *9*, 602–612.
82. Burns, J.A., Weiner, A.B., Catalona, W.J., Li, E.V., Schaeffer, E.M., Hanauer, S.B., Strong, S., Burns, J., Hussain, M.H.A., and Kundu, S.D. (2019). Inflammatory Bowel Disease and the Risk of Prostate Cancer. Eur. Urol. *75*, 846–852.
83. Meyers, T.J., Weiner, A.B., Graff, R.E., Desai, A.S., Cooley, L.F., Catalona, W.J., Hanauer, S.B., Wu, J.D., Schaeffer, E.M., Abdulkadir, S.A., et al. (2020). Association between inflammatory bowel disease and prostate cancer: A large-scale, prospective, population-based study. Int. J. Cancer *147*, 2735–2742.
84. Qiao, X.-R., Zhang, X., Mu, L., Tian, J., and Du, Y. (2020). GRB2-associated binding protein 2 regulates multiple pathways associated with the development of prostate cancer. Oncol. Lett. *20*, 99.
85. Cooperberg, M.R., Zheng, Y., Faino, A.V., Newcomb, L.F., Zhu, K., Cowan, J.E., Brooks, J.D., Dash, A., Gleave, M.E., Martin, F., et al. (2020). Tailoring Intensity of Active Surveillance for Low-Risk Prostate Cancer Based on Individualized Prediction of Risk Stability. JAMA Oncol. *6*, e203187.
86. Xu, J., Isaacs, W.B., Mamawala, M., Shi, Z., Landis, P., Petkewicz, J., Wei, J., Wang, C.-H., Resurreccion, W.K., Na, R., et al. (2021). Association of prostate cancer polygenic risk score with number and laterality of tumor cores in active surveillance patients. Prostate *81*, 703–709.

HGGA, Volume *3*

Supplemental information

Genetic factors associated with prostate cancer conversion from active surveillance to treatment

Yu Jiang, Travis J. Meyers, Adaeze A. Emeka, Lauren Folgosa Cooley, Phillip R. Cooper, Nicola Lancki, Irene Helenowski, Linda Kachuri, Daniel W. Lin, Janet L. Stanford, Lisa F. Newcomb, Suzanne Kolb, Antonio Finelli, Neil E. Fleshner, Maria Komisarenko, James A. Eastham, Behfar Ehdaie, Nicole Benfante, Christopher J. Logothetis, Justin R. Gregg, Cherie A. Perez, Sergio Garza, Jeri Kim, Leonard S. Marks, Merdie Delfin, Danielle Barsa, Danny Vesprini, Laurence H. Klotz, Andrew Loblaw, Alexandre Mamedov, S. Larry Goldenberg, Celestia S. Higano, Maria Spillane, Eugenia Wu, H. Ballentine Carter, Christian P. Pavlovich, Mufaddal Mamawala, Tricia Landis, Peter R. Carroll, June M. Chan, Matthew R. Cooperberg, Janet E. Cowan, Todd M. Morgan, Javed Siddiqui, Rabia Martin, Eric A. Klein, Karen Brittain, Paige Gotwald, Daniel A. Barocas, Jeremiah R. Dallmer, Jennifer B. Gordetsky, Pam Steele, Shilajit D. Kundu, Jazmine Stockdale, Monique J. Roobol, Lionne D.F. Venderbos, Martin G. Sanda, Rebecca Arnold, Dattatraya Patil, Christopher P. Evans, Marc A. Dall'Era, Anjali Vij, Anthony J. Costello, Ken Chow, Niall M. Corcoran, Soroush Rais-Bahrami, Courtney Phares, Douglas S. Scherr, Thomas Flynn, R. Jeffrey Karnes, Michael Koch, Courtney Rose Dhondt, Joel B. Nelson, Dawn McBride, Michael S. Cookson, Kelly L. Stratton, Stephen Farriester, Erin Hemken, Walter M. Stadler, Tuula Pera, Deimante Banionyte, Fernando J. Bianco Jr., Isabel H. Lopez, Stacy Loeb, Samir S. Taneja, Nataliya Byrne, Christopher L. Amling, Ann Martinez, Luc Boileau, Franklin D. Gaylis, Jacqueline Petkewicz, Nicholas Kirwen, Brian T. Helfand, Jianfeng Xu, Denise M. Scholtens, William J. Catalona, and John S. Witte

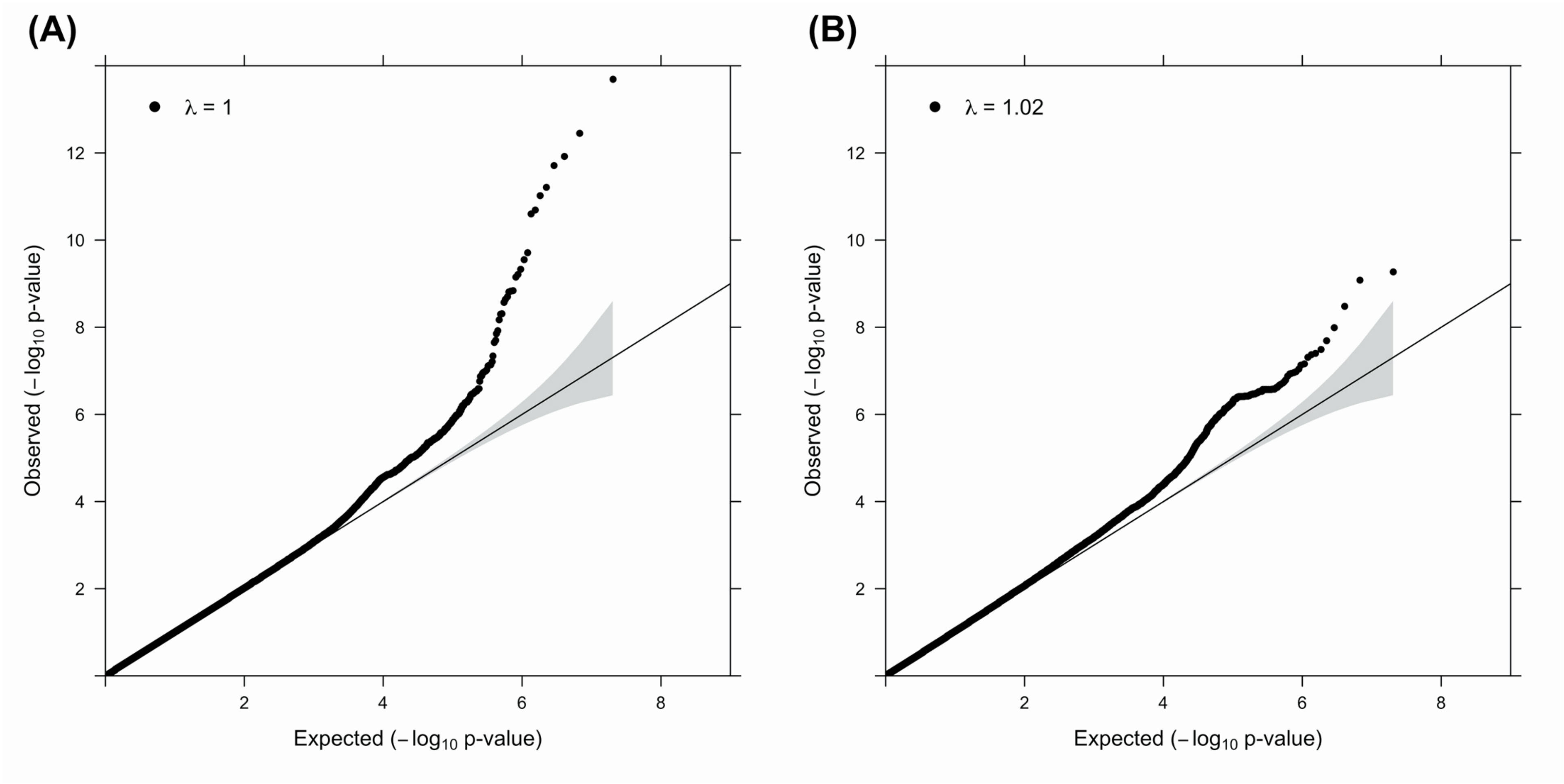

Figure S1. QQ plots of the GWASs. (A) QQ plot of GWAS in individuals of European ancestry. (B) QQ plot of combined GWAS meta-analysis of all individuals. Genomic control λ is shown.

**Discovery Samples**		
Restriction	N Excluded	Total N
Genotyped at CIDR		6,324
independent individuals	23	6,301
With > 6 months follow-up or no missing follow-up	344	5957
With no missing age/censoring status	21	5936
Genetically European	714	5,222
Final N in discovery samples		5,222

**Replication Samples**		
Restriction	N Excluded	Total N
Genotyped at MD Anderson		593
With >6 months follow-up or no missing follow-up	72	591
Non-censored	37	484
Genetically European	59	425
Non-European from CIDR		714
Final N in replication samples		1,139

Table S1: Summary of subjects included and excluded from the discovery GWAS and replication for the study of active surveillance conversion to treatment.

	Discovery		Replication							
Ancestral population	European		African		Asian		Latin American		European[a]	
Sample size	5,222		396		237		81		425	
Mean age (± s.d.)	63.5	(±7.2)	62	(±8.2)	63.8	(±7.7)	63	(±7.2)	64.4	(±8.2)
Median follow-up time (months, IQR)	80	(51,112)	68	(40,106)	86	(54,118)	59	(33,85)	80	(31,110)
Gleason grade										
GG1	4,819	(92%)	359	(91%)	219	(92%)	77	(95%)	372	(87%)
GG2	344	(7%)	32	(8%)	15	(6%)	4	(5%)	48	(11%)
≥ GG3	55	(1%)	4	(1%)	3	(1%)	0	(0%)	5	(1%)
PSA (MAD)	5.0	(±2.1)	5.3	(±2.0)	5.3	(±2.5)	4.7	(±2.4)	4.1	(±2.2)
Clinical tumor stage										
cT1	4,138	79%	322	81%	161	68%	66	81%	372	88%
cT2	641	12%	49	12%	39	16%	9	11%	53	12%
cT3 or cT4	34	1%	5	1%	5	2%	1	1%	0	0%
Number of cores										
1-2	4,113	(79%)	298	(75%)	177	(75%)	67	(83%)	386	(91%)
3	451	(9%)	44	(11%)	20	(8%)	7	(9%)	27	(6%)
≥ 4	522	(10%)	39	(10%)	37	(16%)	7	(9%)	12	(3%)
PC risk category										
Low	3,639	(70%)	262	(66%)	141	(59%)	59	(73%)	314	(74%)
Intermediate	983	(19%)	82	(21%)	53	(22%)	14	(17%)	91	(21%)
High	599	(11%)	52	(13%)	43	(18%)	8	(10%)	20	(5%)
Number of conversions	1609		123		88		21		77	
Tumor upgrading	920	(57%)	68	(55%)	51	(58%)	13	(62%)	44	(57%)
Tumor volume progression	147	(9%)	10	(8%)	6	(7%)	0	(0%)	53	(69%)

PSA Progression	219	(14%)	20	(16%)	15	(17%)	0	(0%)	3	(4%)
Other Reason	134	(8%)	14	(11%)	4	(5%)	2	(10%)	4	(5%)
No Reason Reported	317	(20%)	21	(17%)	20	(23%)	6	(29%)	9	(12%)

Table S2. Characteristics of active surveillance patients included in the discovery and replication genome-wide association study.

a. MD Anderson samples of European Ancestry.

IQR, interquartile range; MAD, median absolute deviation; PSA, prostate-specific antigen; PC, prostate cancer.

Men of genetically inferred European ancestry genotyped by CIDR are included in the discovery GWAS. The other participants genotyped by CIDR and men from MD Anderson are included in the replication.

Age and cancer clinical characteristics were measured at diagnosis.

PC risk categories:

Low-risk patients had all the following criteria: GG1 only (Gleason ≤ 3+3), PSA <10 ng/mL, clinical stage T1, and ≤ 3 positive biopsy cores.

Intermediate-risk patients had any of the following, with no high-risk criteria: GG2 (Gleason 3+4), PSA 10-20 ng/mL, or clinical stage T2.

High-risk patients had any of the following: ≥ GG3 (≥ Gleason 4+3), PSA ≥20 ng/mL, clinical stage ≥T3, or ≥ 4 positive biopsy cores.

Percentages do not all sum to 100% due to missingness.

Model	GRS_{PC}[d]			GRS_{PSA}[d]			AUC
	HR	95% CI	P-value	HR	95% CI	P-value	
Reference model[a]							0.550
PC clinical characteristics[b]							0.653
PC GRS[c]	1.18[e]	1.12, 1.23	5.6×10^{-11}				0.576
PSA GRS[c]				0.92[e]	0.88, 0.96	3.9×10^{-4}	0.557
PC clinical characteristics + PC GRS[c]	1.13[e]	1.07, 1.19	3.3×10^{-6}				0.659
PC clinical characteristics + PSA GRS[c]				0.94[e]	0.89, 0.98	8.5×10^{-3}	0.655
PC clinical characteristics + PC GRS + PSA GRS	1.15[e]	1.09, 1.22	1.5×10^{-7}	0.91[e]	0.87, 0.96	3.0×10^{-4}	0.661

Table S3. **ROC analysis of PC and PSA GRS compared to clinical characteristics for conversion from AS to treatment.**

[a] All models contain age and the first 10 principal components.

[b] PC clinical characteristics were Gleason grade group (GG1, GG2, or ≥ GG3); PSA concentration (ng/mL); clinical stage (cT1, cT2, or cT3/cT4); and number of positive biopsy cores (1-2, 3, or ≥ 4).

[c] PC GRS is constructed from 269 prostate cancer associated variants, while PSA GRS is derived from 36 PSA risk variants.

[d] The test statistics of GRS_{PC} and GRS_{PSA} are reported when the model contains them.

[e]The HRs correspond to a one SD increase in the GRS.

	Minimally Adjusted			Fully Adjusted		
	HR[a]	95% CI	P	HR[b]	95% CI	P
**Decile**						
0-10	0.73	0.60, 0.89	0.0016	0.69	0.56, 0.86	0.0010
10-20	0.81	0.68, 0.98	0.030	0.83	0.68, 1.01	0.067
20-30	0.93	0.78, 1.11	0.44	0.99	0.82, 1.20	0.95
30-40	0.83	0.69, 0.99	0.041	0.82	0.67, 0.99	0.043
40-60	1.00	Reference		1.00	Reference	
60-70	1.06	0.89, 1.26	0.50	1.03	0.86, 1.24	0.72
70-80	1.10	0.92, 1.30	0.30	1.02	0.85, 1.22	0.87
80-90	1.23	1.04, 1.46	0.014	1.15	0.96, 1.37	0.13
90-100	1.27	1.07, 1.51	0.0061	1.13	0.94, 1.36	0.18

Table S4. Hazard ratios for the association between time to AS failure with prostate cancer GRS.
HR = Hazard Ratio; CI = Confidence Interval

[a] Hazard ratios are adjusted for age and the first 10 genetic principal components

[b] Hazard ratios are adjusted for age, the first 10 genetic principal components, Gleason grade group (GG1, GG2, or ≥ GG3); PSA concentration (ng/mL); clinical stage (cT1, cT2, or cT3/cT4); and number of positive biopsy cores (1-2, 3, or ≥ 4).

	Minimally Adjusted			Fully Adjusted		
	HR[a]	95% CI	P	HR[b]	95% CI	P
**Decile**						
0-10	1.28	1.08, 1.52	0.0044	1.25	1.04, 1.50	0.017
10-20	1.08	0.90, 1.29	0.40	1.01	0.83, 1.22	0.96
20-30	1.15	0.97, 1.37	0.11	1.18	0.98, 1.42	0.083
30-40	1.16	0.98, 1.38	0.088	1.16	0.96, 1.39	0.12
40-60	1.00	Reference		1.00	Reference	
60-70	1.08	0.90, 1.29	0.40	1.05	0.86, 1.27	0.65
70-80	1.00	0.83, 1.19	0.96	0.96	0.79, 1.17	0.70
80-90	0.98	0.82, 1.18	0.84	1.02	0.84, 1.24	0.87
90-100	0.95	0.79, 1.15	0.60	0.97	0.79, 1.19	0.78

Table S5. Hazard ratios for the association between time to AS failure with prostate-specific antigen GRS.

HR = Hazard Ratio; CI = Confidence Interval

[a] Hazard ratios are adjusted for age and the first 10 genetic principal components

[b] Hazard ratios are adjusted for age, the first 10 genetic principal components, Gleason grade group (GG1, GG2, or ≥ GG3); PSA concentration (ng/mL); clinical stage (cT1, cT2, or cT3/cT4); and number of positive biopsy cores (1-2, 3, or ≥ 4).
